# Light-triggered photosynthetic engineered bacteria for enhanced-photodynamic therapy by relieving tumor hypoxic microenvironment

**DOI:** 10.7150/thno.81718

**Published:** 2023-03-13

**Authors:** Chenyang Yin, ZeKun Wang, Chunxue Dai, Bangjia Yang, Weiyun Wang, Endong Yang, Feng Guo, Cundong Fan, Pu Zhang, Jikui Sun, Dongdong Sun

**Affiliations:** 1 School of Life Sciences, Anhui Agricultural University, Hefei 230036, China.; 2 Shandong Key Laboratory of TCM Multi-Target Intervention and Disease Control, the Second Affiliated Hospital of Shandong First Medical University, Taian, Shandong 271000, China.; 3 Department of Cardiology, Affiliated Taian City Central Hospital of Qingdao University, Taian, Shandong, 271000, China.; 4 Department of Neurosurgery, the First Affiliated Hospital of Shandong First Medical University & Shandong Provincial Qianfoshan Hospital, Jinan, China.

**Keywords:** singlet oxygen, photodynamic therapy, tumor hypoxic microenvironment, photosynthetic bacteria, photothermal therapy.

## Abstract

**Background:** Singlet oxygen (^1^O_2_) has received considerable research attention in photodynamic therapy (PDT) due to its cytotoxic solid features. However, the inherent hypoxic state of the tumor microenvironment (TME) leads to the meager ^1^O_2_ quantum yield of inorganic PDT reagents, and their application *in vivo* remains elusive.

**Methods:** We developed a novel strategy to fabricate active photosynthetic bacteria/photosensitizer/photothermal agent hybrids for photosynthetic tumor oxygenation and PDT and PTT tumor therapy under different laser irradiation sources. Photosynthetic bacteria combined with Ce6 photosensitizer and Au NPs photothermal agent, the obtained Bac@Au-Ce6 effectively targets tumor tissues and further enhances the tumor accumulation of Au-Ce6.

**Results:** The results showed that the Au-Ce6-loaded engineered bacteria (Bac@Au-Ce6) maintained the photosynthetic properties of *Syne*. After i.v. injection, Bac@Au-Ce6 efficiently aggregates at tumor sites due to the tumor-targeting ability of active *Syne*. With 660 nm laser irradiation at the tumor site, the photoautotrophic *Syne* undergoes sustained photosynthetic O_2_ release and immediately activates O_2_ to ^1^O_2_ via a loaded photosensitizer. PTT was subsequently imparted by 808 laser irradiations to enhance tumor killing further.

**Conclusions:** This work provides a new platform for engineering bacteria-mediated photosynthesis to promote PDT combined with PTT multi-faceted anti-tumor.

## Introduction

The tumor microenvironment (TME) is often characterized by tumor hypoxia, with a total partial pressure of oxygen (pO_2_) below 20 mmHg 1, 2, 3, 4, 5, 6, 7. Local hypoxia in tumors seriously hinders the treatment of cancer, resulting in significantly reduced efficacy, especially for photodynamic clinical therapy (PDT) 8, 9, 10, 11, therefore, adequate tumor oxygenation is attractive for studying this clinically promising antitumor approach. According to past reports, FDA-approved perfluorocarbon (PFC) materials (such as C5F12) are used to transport oxygen molecules 12 directly, but PFC-based tumor oxygenation strategies are difficult to deliver enough oxygen for PDT therapy. Problems that lead to the inefficiency of the drug's action have so far remained unresolved, worsening photodynamic therapy outcomes. Chemical oxygen-releasing nanomaterials (*e.g.*, MnO_2_ nanoparticles 13, 14, 15 are designed to couple PDT systems for tumor therapy. Quantitative O_2_ production performance can guarantee valid PDT results. However, toxic ionic products such as Mn^2+^ raise potential biocompatibility issues. Biocatalysts (*e.g.*, catalase 16 have also been designed to improve tumor hypoxia to optimize PDT, but biocatalysts readily maintain stable activity in complex physiological environments. Therefore, it becomes of great interest to develop a method to fundamentally improve and fabricate a sustained, biocompatible, and efficient oxygenated photodynamic system for tumor therapy.

Bacteria have many advantages in cancer therapy, such as the ease of growth of natural bacteria, the low cost of culture, and the inherent tumor-targeting ability. Numerous studies have exploited the tumor-targeting capabilities of bacteria for targeted drug release or delivery of various functional nanoparticles to tumors for precise tumor suppression 17, 18, 19. Photosynthetic bacteria with photoautotrophic ability can utilize chlorophyll molecules on thylakoid membranes for oxygen-evolving photosynthesis, and photo-controllable growth and photosynthesis are of particular interest. Cyanobacteria are natural photosynthetic bacteria that provide vital oxygen molecules during the emergence and evolution of life on earth 20. Based on the oxygen evolution properties of cyanobacteria, it has been designed and explored for the treatment of cardiovascular and cerebrovascular diseases 21. Nevertheless, there are currently very few reports on the use of microorganism-based biological tumor oxygenation for tumor therapy. Therefore, the application of photosynthetic bacteria is a promising approach to alleviate tumor hypoxia and promote PDT.

After a long period of research, various types of bacterial cells have been designed as a delivery vehicle for the delivery of chemotherapeutic drugs 22 plasmids 23 proteins 24, and other composite nanomedicines for tumor therapy 25. This composite nanosystem can endow bacterial cells with multiple therapeutic functions, and can comprehensively utilize a variety of methods for multi-directional treatment, such as Fenton noncatalytic therapy 26, photothermal eradication 27, and biological therapy 28. Photothermal therapy (PTT) utilizes photothermal agents to convert light into heat energy under NIR irradiation at specific wavelengths and kill tumor cells in an O_2_-independent manner. PDT and PTT both kill cells by inducing free radical production. However, both PTT and PDT have their inevitable defects, and it is difficult to achieve a full therapeutic effect under a single mode. PTT and PDT combined therapy not only inherits the advantages of light therapy with low toxicity and few side effects but also can make the two treatments result in additional and even synergistic therapeutic effects, which is an effective strategy to improve curative effect and reduce toxic side effects 29, 30, 31, 32.

Despite the tremendous medical potential achieved, key challenges such as surface interactions, device degradation, and *in vivo* toxicity exist, which limits their clinical application 33. Therefore, there is a great need to design newly engineered bacteria with greater biocompatibility for effective cancer diagnosis and treatment.

**Scheme 1:**
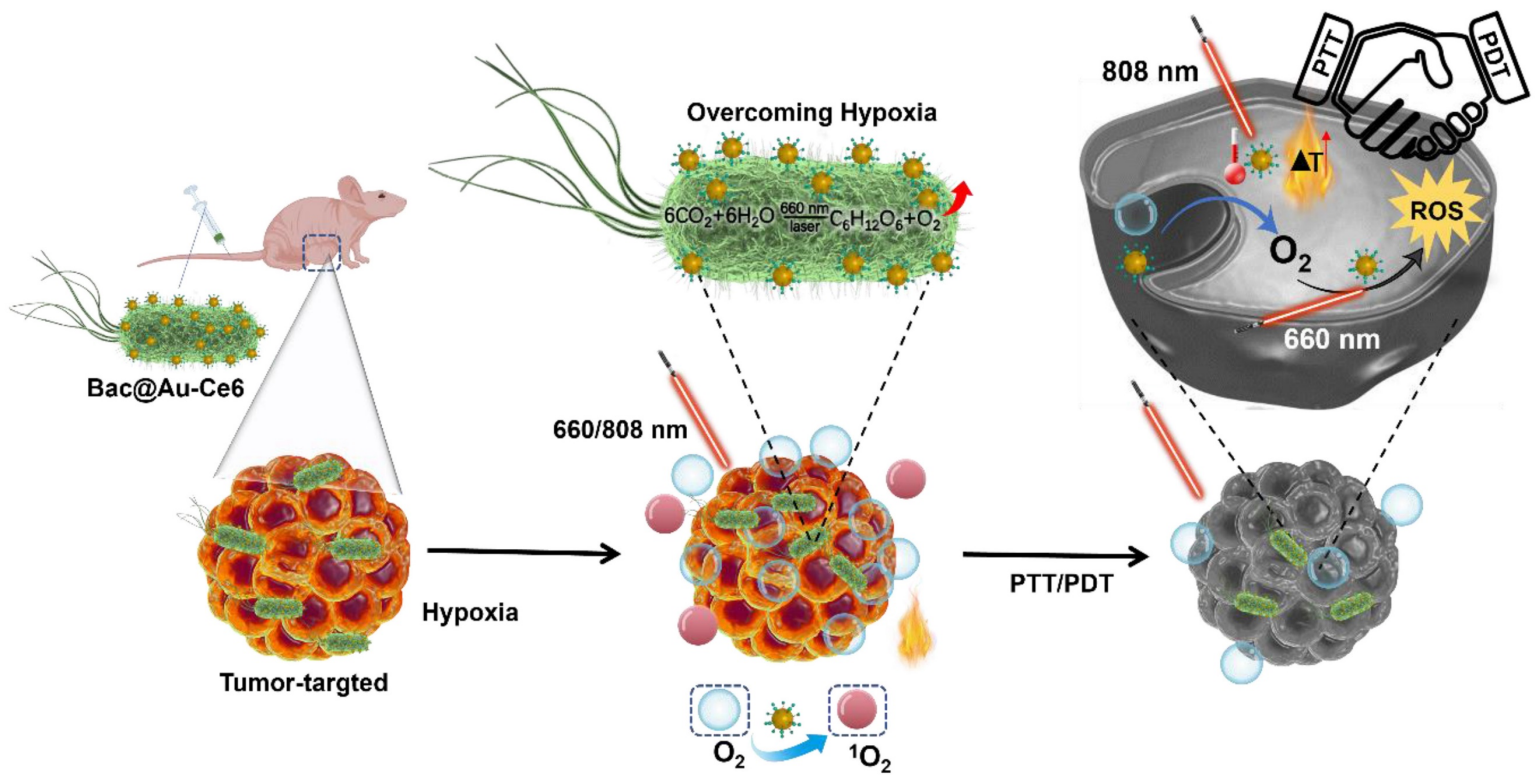
Synthesis of Bac@Au-Ce6 and schematic diagram of PDT and PTT tumor therapy in vivo.

In this study, we utilized *Synechococcus* 7942 (*Syne*) a native photosynthetic bacterium and combined it with photosensitizer Ce6-encapsulated Au NPs nanoparticles (Au-Ce6) for 4T1 tumor-bearing mice Treatment (Scheme 1). The tumor-targeting ability of photosynthetic *Syne* transports Au-Ce6 to the tumor, and increases tumor oxygen concentration through photosynthetic oxygenation, thereby enhancing the PDT effect. The results showed that the Au-Ce6-loaded engineered bacteria (Bac@Au-Ce6) maintained the photosynthetic properties of *Syne*. After i.v. injection, Bac@Au-Ce6 efficiently aggregates at tumor sites due to the tumor-targeting ability of active *Syne* 34, 35, 36. With 660 nm laser irradiation at the tumor site, the photoautotrophic *Syne* undergoes sustained photosynthetic O_2_ release and immediately activates O_2_ to ^1^O_2_ via a loaded photosensitizer. PTT was subsequently imparted by 808 laser irradiations to enhance tumor killing further. This work provides a new platform for engineering bacteria-mediated photosynthesis to promote PDT combined with PTT multi-faceted anti-tumor.

## Experimental

### Materials and bacterial strain

Chloroauric acid (HAuCl_4_·3H_2_O), Polyethyleneimine (PEI, Mw 10,000), singlet oxygen sensor green reagent (SOSG), 3-(4,5-Dimethylthiazol-2-yl)-2,5-diphenyltetrazolium bromide (MTT) and Chlorine6 were all purchased from Aladdin. 4T1 murine breast tumor cells were purchased from the Cell Bank of Type Culture Collection of Chinese Academy of Sciences (Shanghai, CHN). 1,3-Diphenylisobenzofuran (DPBF) was purchased from Sigma-Aldrich. All chemicals were used without further purification. Phosphate buffered saline (PBS): 0.1 M NaCl + 20 mM phosphate buffer, adjusted to pH 7.4. All solutions were prepared using filtered and UV-treated ultrapure water with a resistivity of 18.2 MΩ.cm (LAB-WP-Extract, LEADING, Shanghai, China). Cyanobacteria *Syne*chococcus elongatus PCC 7942 strain was obtained from Freshwater Algae Culture Collection at the Institute of Hydrobiology (FACHB). *Syne* was cultured with BG11 medium at 25 °C under the light. The inductively coupled plasma-mass spectrometer (ICP-MS) was taken on an Icap 6300 of Thermo Scientific.

### Preparation and characterization of Au-Ce6-loaded *Syne*

An aqueous solution of HAuCl_4_·3H_2_O (2.54 M, 18 μL) was added to deionized water (30 mL) that had been heated to boiling point (100 °C). Sodium citrate solution (3 wt%, 1 mL) was then added and the resulting mixture was stirred for 20 min. PEI (12.8 mg/mL, Mw 10,000, 100 μL) was added and stirred for 2 h, then 100 μL of Ce6 (2,500 μg/mL in DMSO) was added, and the resulting mixture was stirred for 1 h. After completion of the reaction, the products were collected (8000 rpm for 4 min) and washed twice with water. The Au-Ce6 NPs product was collected as an aqueous dispersion. For Au-Ce6 NPs loading, *Syne* was mixed with the Au-Ce6 NPs for 30 min at 25 °C. Finally, Bac@Au-Ce6 was collected from unbound Au-Ce6 by centrifugation and washed with PBS.

Scanning electron micrograph (SEM, S-4800, Hitachi Co. Ltd., Japan) and Transmission electron micrograph (TEM, HT-7700, Hitachi Co. Ltd., Japan) were applied to identify the morphology of Au-Ce6-loaded *Syne*. The absorption spectra were measured with a UV-vis spectrometer (UV-3600i Plus, SHIMADZU CO., LTD), and the fluorescence spectra were recorded via fluorescence spectroscopy (Edinburgh FS920, UK). The hydrodynamic diameters of Au-Ce6 NPs and Bac@Au-Ce6 were determined by dynamic light scattering (DLS, Malvern Zetasizer, Nano-ZS90, UK).

### Oxygen production by photosynthesis was measured *in vitro*

Bac@Au-Ce6 (6 × 10^8^ in 8 mL BG11 medium) were exposed to different light or dark conditions. O_2_ production was measured using an oxygen probe (JPBJ-608 portable dissolved oxygen meter) and the dissolved oxygen value was recorded at predetermined times. The air is taken out by a vacuum pump and CO_2_ was added to restore the original pressure. Oxygen production was then measured using the above method.

### Detection of singlet oxygen generation

The production of singlet oxygen was detected by DPBF (as a molecular probe). Specifically, 20 μL of DPBF (20 mg/mL in DMF) in PBS buffer, *Syne* (2 × 10^7^ CFU), Au-Ce6 (3 μg of Ce6 concentration), and Bac@Au-Ce6 (2 × 10^7^ *Syne* corresponding to 3 μg of Ce6 concentration) were treated with a 660 nm (20 mW/cm^2^) and an 808 nm laser (2 W/cm^2^) irradiation for a fixed time. Changes in the DPBF concentration were recorded by monitoring max = 420 nm in the UV-vis spectra. In addition, the production of ^1^O_2_ was detected by the molecular probe SOSG. Briefly, Bac@Au-Ce6 and SOSG were mixed and exposed to laser irradiation (660 nm, 20 mW/cm^2^ and 808 nm laser, 2 W/cm^2^) at fixed intervals durations. A fluorescence spectrophotometer was used to detect the fluorescence intensity of SOSG.

### *In vitro* cytotoxicity assay

The cytotoxicity of Bac@Au-Ce6 against various cell lines (4T1, SKOV3, HEK293, HepG2, fibroblasts, Jurkat T, and J774 cells) was determined by standard thiazolyl tetrazolium (MTT). Briefly, cells were seeded in 96-well plates at a density of 1 × 10^4^ cells per well, incubated at constant temperature for 12 h, and incubated at different concentrations of Bac@Au-Ce6 (*Syne* = 1 × 10^6^, 2 × 10^7^, 2 × 10^8^,1 × 10^9^, 2 × 10^9^ correspond 3 μg, 3 μg, 6 μg, 20 μg, 30 μg of Ce6 concentration, respectively) for at least 24 h. Thereafter, the cell culture medium was gently rinsed with PBS, then 20 μL of MTT solution (5 mg/mL) was added to each well and incubated for an additional 4 h at 37 °C for assay.

For the hemolysis assay, 5 mL of mouse blood was centrifuged (1800 rpm, 12 min) and washed 5 times with PBS to collect RBCs. Diluted RBC suspensions with four volumes of different concentrations of dispersion (in PBS) at different concentrations of Bac@Au-Ce6 (*Syne* = 1 × 10^6^, 2 × 10^7^, 2 × 10^8^, 1 × 10^9^, 2 × 10^9^ correspond to 3 μg, 3 μg, 6 μg, 20 μg, 30 μg of Ce6 concentration, respectively) mixed. Deionized water is a positive control. After 5 h incubation, all mixed suspensions were centrifuged (13,000 rpm, 15 min) and optical density at 450 nm was determined by a microplate reader (Molecular Devices, USA).

### Photothermal property *in vitro* and drug release

To evaluate the photothermal effect of Bac@Au-Ce6 under 808 nm laser irradiation, Bac@Au-Ce6 (dispersed in PBS) was irradiated with laser light (2 Wcm^-2^) for 10 min. The temperature and thermographic images of the Bac@Au-Ce6 dispersion during irradiation were recorded using an infrared thermal imager (Fluke TiR27, USA). Besides, to evaluate the photothermal stability of Bac@Au-Ce6, the Bac@Au-Ce6 dispersion was laser irradiated under the same conditions, and the temperature changes during 4 cycles were recorded. The release behavior of Au-Ce6 from Bac@Au-Ce6 was investigated using the dialysis method. First, 2 mL of Bac@Au-Ce6 (dispersed in PBS) was placed in a dialysis bag (3500 D), With or without laser irradiation, then maintained at 37 °C in a bath of phosphate-buffered saline (PBS) (pH 7.4) solution with constant stirring. Samples were retrieved at the indicated time points (1, 2, 4, 8, 16, 48, 96, 144, 216, 336 h) to measure the absorbance at 404 nm using an ultraviolet-visible (UV-vis) spectrophotometer.

Ce6 Drug loading efficiency (%) = (weight of Ce6 loaded)/ (total weight of Au-Ce6) × 100%.

Au-Ce6 Drug loading efficiency (%) = (weight of Au-Ce6 loaded)/ (total weight of Bac@Au-Ce6) × 100%.

### Cellular uptake of Bac@Au-Ce6 nanocomposites

Bac@Au-Ce6 was first mixed with HS-PEG-NH_2_ for 8 h to form amino-modified Bac@Au-Ce6. RhB (2 mg) was dissolved in PBS buffer (2 mL, pH 7.4), and 5 mg of EDC and 10 mg of NHS (both in 3 mL PBS, pH 7.4) were added, and the resulting solution was stirred in the dark for 2 h. Subsequently, the amino-modified Bac@Au-Ce6 was added to this solution with stirring for a further 10 h in the dark. The resulting product was centrifuged at 3000 rpm for 3 min and washed with PBS (3 mL, pH 7.4) 3 times.

### *In vitro* cellular oxygenation assay

The [Ru(dpp)_3_]Cl_2_ (RDPP) probe was used to monitor intracellular O_2_ production. 4T1 tumor cells were initially seeded into six-well plates at a cell density of 10^5^ cells/well. After incubation for 12 h at a constant temperature, 4T1 cells were pretreated with a hypoxic mimetic (deferoxamine, DFO) for 12 h in a hypoxic incubator under a hypoxic atmosphere (1% O_2_, 5% CO_2_, and 94% N_2_). Cells were then sealed and incubated with RDPP (10 μg/mL in complete DMEM medium) for 3 h (DFO removed). 1 mL full DMEM medium containing *Syne* (*Syne* = 2×10^7^), Au-ce6 (3 μg of Ce6 concentration), Bac@Au-Ce6 (*Syne* = 2×10^7^, 3 μg of Ce6 concentration) was supplemented to replace the previous medium after mild rinsing with cold PBS respectively. Cells were treated with or without 660 nm laser irradiation (20 mW/cm^2^, 10 min) and observed under a confocal microscope (Ex: 488 nm, Em: 610 nm).

### *In vitro* cellular SOSG Detection

The *in vitro* cellular singlet oxygen generation detection was realized by the cellular SOSG assay. 4T1 tumor cells were initially seeded into six-well plates at a cell density of 10^5^ cells/well. before the supplementations of *Syne* (*Syne* = 2×10^7^), Au-Ce6 (3 μg of Ce6 concentration), Bac@Au-Ce6 (*Syne* = 2×10^7^, 3 μg of Ce6 concentration) containing full medium. After the co-incubation for 2.5 h, the singlet oxygen probe SOSG was directly added to incubate with the tumor cells for 10 min. With or without 660 nm laser (20 mW/cm^2^) treated cells for 10 min. Immediately, the medium was discarded, and cells were stained 4',6-diamidino-2-phenylindole (DAPI) for 10 min.

### *In vitro* cellular PDT and PTT experiment

L929 fibroblast cells and 4T1 tumor cells were seeded into 96-well plates at a density of 10^4^ cells/well. Incubate at constant temperature for 12 h. The previous medium was then replaced with a fresh medium containing PBS, *Syne*, Au-Ce6, or Bac@Au-Ce6 after a gentle rinse with PBS. With a co-incubation time of 2 h, 660 nm laser (20 mW/cm^2^) and 808 nm laser (2 W/cm^2^) were applied to irradiate for 10 min. After an additional 12 h of co-incubation, proceed to typical cell counting kit procedures. Absorbance at 490 nm was measured to indicate relative cell viability relative to controls.

### Tumor model

Female Balb/c mice (∼20 g, 6-8 weeks old) were used as a tumor model in animal experiments and were purchased from Nanjing Zi yuan Biotechnology Co., Ltd. All animal experiments were carried out according to the protocols approved by Anhui Agricultural University Laboratory Animal Center.

### *In vivo* PA imaging, PTT performance, and *in vivo* fluorescence imaging

To evaluate *in vivo* PA imaging properties, Bac@Au-Ce6 (100 μL suspended in PBS, a dose of Ce6 = 0.15 mg/kg and *Syne* = 1 × 10^9^/kg) was injected intravenously (i.v.) of tumor-bearing Balb/c mice. At 0, 0.5, 2, 4, and 8 h after i.v. injection, the tumor site was imaged by the MSOT system (inSight/ inVision 256, iTheramedical, Germany). Selected wavelength: 680 nm; step size of imaging: 1 nm. All results were analyzed using the View MSOT software suite included with the MSOT system. Another group of tumors bearing BALB/c mice injected with Bac@Au-Ce6 (100 μL suspended in PBS, dose of Ce6 = 0.15 mg/kg and *Syne* = 1 × 10^9^/kg), followed by irradiated (2 W/cm^2^, 808 nm) for 10 min. Then the mice were photographed with an infrared thermal imaging camera (Fluke TiR27, USA) at 2-min intervals. 4T1 tumor-bearing Balb/c mice were i.v. injected with Bac@Au-Ce6 (100 μL suspended in PBS, dose of Ce6 = 0.15 mg/kg and *Syne* = 1 × 10^9^/kg) and then photographed by IVIS Lumina LT Series III (Perkin Elmer, Massachusetts, USA) at different time points including 2, 4, 8 h post-injection.

### *In vivo* antitumor effect

Twenty female BALb/c nude mice were separated into five groups randomly. Mice were injected subcutaneously with 4T1 breast cancer cells, and when tumors grew to approximately 100 mm^3^, mice were i.v. injected with PBS, *Syne*, Au-Ce6 or Bac@Au-Ce6 (dose of Ce6 = 0.15 mg/kg and *Syne* = 1 × 10^9^/kg), respectively. The 808 nm laser (2 W/cm^2^, 10 min) and 660 nm laser (50 mW/cm^2^, 10 min) irradiation was performed at 4 h after injection. Tumor volume was measured every two days. Tumor volume was calculated according to this formula: *volume=width^2^* ×*length*/*2*. (All group of the administration volume is 100 μL).

Tumor tissues were collected and analyzed on the 14th day after different treatments by immunolocalizing HIF-1α (hypoxiainducible factor 1-alpha), SOSG, PCNA (proliferating cell nuclear antigen), Caspase-3, and terminal deoxynucleotidyl transferase dUTP nick end labeling (Tunel). We also conducted hematoxylin and eosin (H&E) staining.

### *In vivo* biocompatibility experiment

For long-term toxicity study, mice (n = 5) were i.v. injected with 100 μL of PBS, Bac@Au-Ce6 (dose of Ce6 = 0.15 mg/kg and *Syne* = 1 × 10^9^/kg), Bac@Au-Ce6 with or without laser irradiation (808 nm laser, 2 W/cm^2^, 10 min and 660 nm laser, 50 mW/cm^2^, 10 min). Sixty days after i.v. injection, the mice were killed, blood samples were collected for blood science and clinical tests, and the major organs (heart, liver, spleen, lung, and kidneys) of mice were harvested for systematical pathological analysis. To explore the *in vivo* bio-distribution of bacteria after each treatment, tissues were collected from mice with tumors including; heart, liver, spleen, lung, and kidney, and these organs were homogenized with 0.1% Triton X-100 on days 1, 3, and 7 to obtain data on the viability of bacteria in each organ.

In the quantitative biodistribution analysis, 4T1 tumor-bearing mice were intravenously injected with Bac@Au-Ce6 (100 μL suspended in PBS, dose of Ce6 = 0.15 mg/kg and *Syne* = 1 × 10^9^/kg). At 2, 4, 8, 24 h, 3, 7 and 14 days post-administration, the mice were sacrificed. The tumor tissues and main organs containing heart, liver, spleen, lung, and kidneys were excised and weighed, then digested in aqua regia under heat treatment (70 °C) for homogenate to analyze the content of Au in the samples using ICP-MS.

## Results and Discussion

### Preparation and Characterization of Au-Ce6-Loaded *Syne*

**Figure 1:**
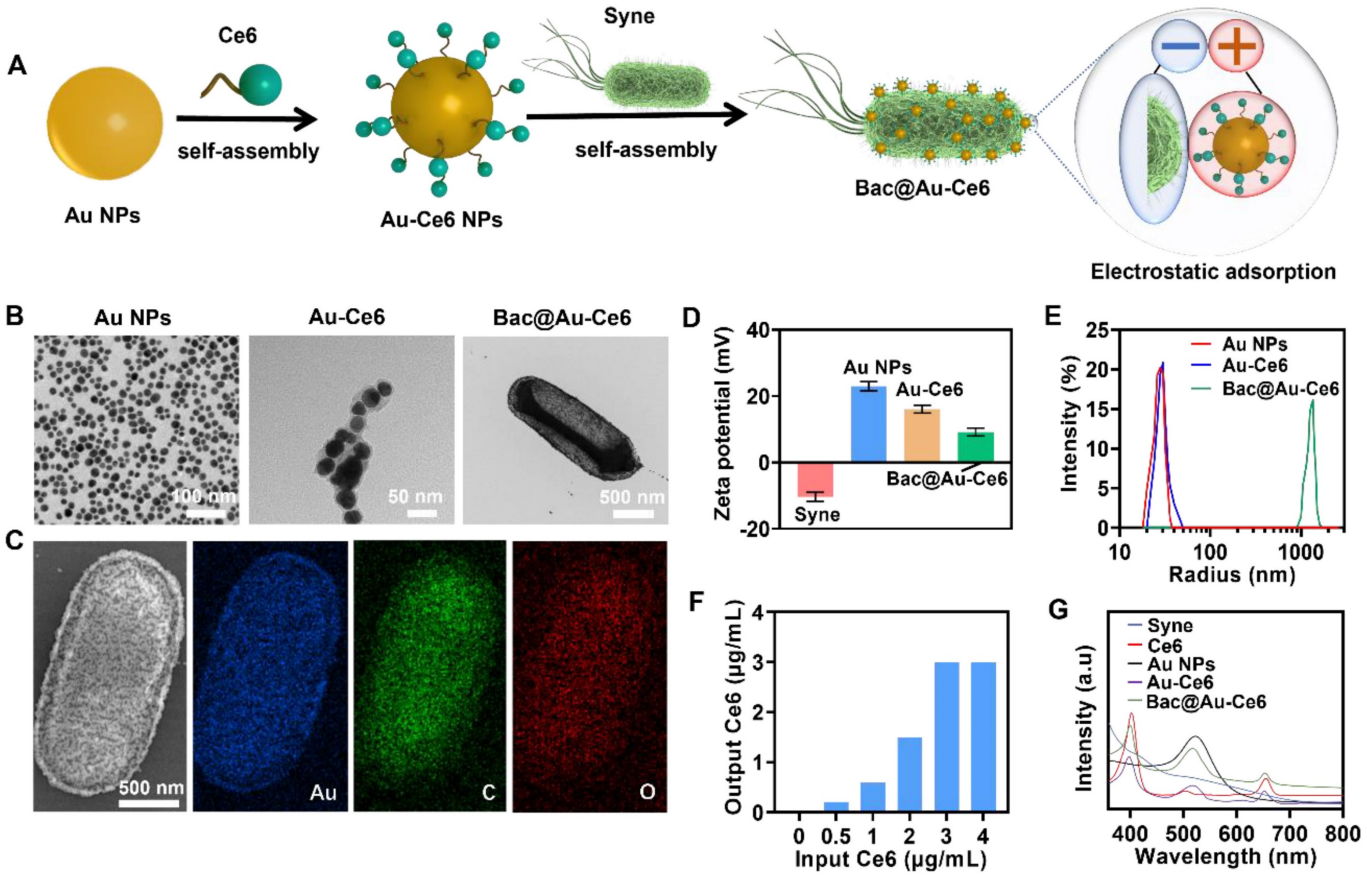
** Preparation and characterization of Au-Ce6-loaded *Syne*. (A)** The synthetic process of Bac@Au-Ce6. **(B)**TEM images of Au NPs, Au-Ce6, and Bac@Au-Ce6. **(C)** SEM image and the corresponding elemental mapping images of Au NPs, Au-Ce6, and Bac@Au-Ce6. **(D)** Zeta potential of Syne, Au NPs, Au-Ce6, and Bac@Au-Ce6 (Mean ± SD, n = 3). **(E)** Dynamic light scattering (DLS) size distribution profiles of Au NPs, Au-Ce6, and Bac@Au-Ce6. **(F)** The relationship between the release concentration and the addition concentration of Ce6 concentration in Bac@Au-Ce6 solution. **(G)** UV-vis spectra of *Syne* cells, Au NPs, free Ce6 photosensitizer, Au-Ce6, and Bac@Au-Ce6.

The fabrication process of Au-Ce6-loaded *Syne* (hereafter Bac@Au-Ce6) was described in Figure 1A. First, nanophotosensitizers (Au-Ce6) were constructed by assembling Au NPs and Ce6 through electrostatic adsorption. The obtained Au-Ce6 NPs were uniformly spherical, and the Au NP core was coated with a Ce6 that had a thickness of about 3 nm. Negatively charged *Syne* adsorbs positively charged Au-Ce6 to form Bac@Au-Ce6. The TEM mapping of Au-Ce6 observed Au elements and the constituent elements of Ce6 (C, H, O, N) (Figure S1). In addition, Ce6 displayed peaks at 1711, 1690, and 1232 cm^-1^, which is assigned to the stretching vibration of C=O, C=N, and C=C. The FTIR spectrum of Au-Ce6 displayed all these three representative peaks of Ce6, verifying the successful synthesis of the final product. (Figure S2). Representative TEM images (Figure 1B) showed that Au-Ce6 NPs were successfully attached to the surface of *Syne*. Moreover, the elemental mapping of Au, O, and C confirmed a uniform distribution of elements due to the NPs in the Bac@Au-Ce6 (Figure 1C). The process of assembly of the nanomedicine via electrostatic adsorption was confirmed by comparisons of the Zeta potential (ξ) which indicates the change in surface charge (Figure 1D). Au-Ce6 NPs (ξ = 22.5 ± 1.3 mV) were attached to the surface of *Syne* (ξ =-10.2 ± 0.5 mV) through electrostatic adsorption to form Bac@Au-Ce6 (ξ =9.3 ± 1.1 mV). The dynamic light scattering (DLS) method provided insight into the changes in particle sizes (Figure 1E).

To improve the adsorption efficiency of Au-Ce6 NPs, *Syne* (2 × 10^7^ CFU) with different feed ratios was incubated with Au-Ce6 (0.5, 1, 2, 3, 4 µg/mL) for 1 h. The results showed that the adsorption rate of Au-Ce6 NPs increased in a dose-dependent manner. The adsorption efficiency (2 × 10^7^ CFU) reached a maximum when the concentration of Au-Ce6 NPs was 3 µg/mL (Figure 1F). Therefore, we selected Au-Ce6 NPs at a concentration of 3 µg/mL for all subsequent experiments and analyses. Furthermore, the loading efficiency of Ce6 on the Au-Ce6 was about 25.73 wt%, and the loading efficiency of Au-Ce6 on the Bac@Au-Ce6 was about 7.26 wt% based on the standard curve of Ce6 (Figure S3). The absorption spectrum of *Syne* shows broad absorption in the UV-Vis range. The UV-Vis spectra of Bac@Au-Ce6 and Au-Ce6 have a characteristic absorption peak of Ce6 at 404 nm and a characteristic peak of Au NPs at 522 nm. These results indicated that Ce6 was successfully adsorbed on Au-Ce6, the additional absorption peak also clearly manifests the successful fabrication of the Bac@Au-Ce6 (Figure 1G).

Next, we examined Bac@Au-Ce6 cell viability, cytotoxicity, alleviation of hypoxia, and enhanced photodynamic therapy ability. Bac@Au-Ce6 showed high survival rates (both higher than 82%) during the 7-day test period in the growth environment (Figure S4A) and in the varying temperature test (Figure S4B). Analysis by methylthiazolyl tetrazolium (MTT) method showed that Bac@Au-Ce6 did not affect a variety of cells (human SKOV3 ovarian Survival of cancer cells, human kidney (HEK) 293 cells, HepG2 cells, fibroblasts, Jurkat T cells (Figure S4C-S4H) or murine J774 macrophages. In the hemolysis experiments, Bac@Au-Ce6 did not cause obvious hemolysis in the experimental concentration range (Figure S4I).

### Cascade Oxygenation and Photosensitization of Bac@Au-Ce6

**Figure 2:**
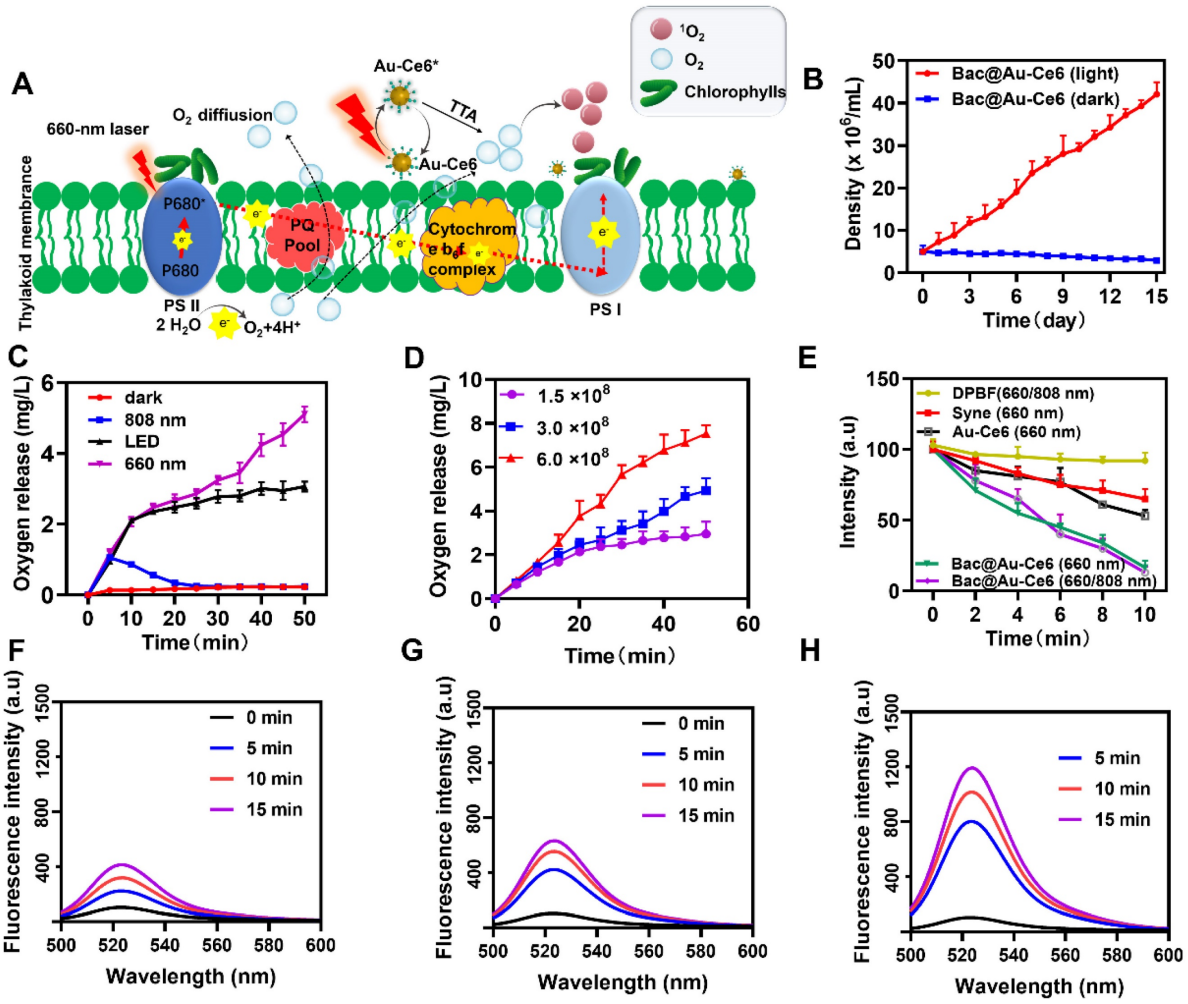
** Photosynthetic O_2_ evolution and photosensitization effect by Bac@Au-Ce6. (A)** Schematic illustration of the photosynthesis-enhanced photodynamic process. **(B)** The growth curve of Bac@Au-Ce6 in the light or dark (Mean ± SD, n = 3). **(C)** Oxygen production curves of Bac@Au-Ce6 under different conditions. LED irradiation with a wavelength range of 400-750 nm (Mean ± SD, n = 3). **(D)** O_2_ production curves of various concentrations of Bac@Au-Ce6 treated with 660 nm laser irradiation (Mean ± SD, n = 3). **(E)** Comparison of DPBF degradation by *Syne*, Au-Ce6 (660 nm), Bac@Au-Ce6 (660 nm), and Bac@Au-Ce6 (660 nm, 808 nm) under laser irradiation for 10 min (Mean ± SD, n = 3). **(F)** Fluorescence intensity profiles of SOSG assays for free *Syne*. **(G)** Fluorescence intensity profiles of SOSG assays for Au-Ce6. **(H)** Fluorescence intensity profiles of SOSG assays for Bac@Au-Ce6. Laser irradiation (20 mW/cm^2^, 660 nm) was applied to trigger the photodynamic process.

Laser irradiation of Bac@Au-Ce6 can induce photosynthetic oxygenation of *Syne* cells therein while sensitizing the loaded Ce6 photosensitizer. According to the photosynthetic biology of *Syne*, under laser irradiation, *Syne* produces a strong photosynthetic oxygenation effect at the photosystem II (PS II) complex within the thylakoid membrane 37. The photosynthesis process begins with the excitation of a chlorophyll molecule (usually P680, a variant of chlorophyll a) in the photosystem II complex (PS II, a unit of photosynthesis in the thylakoid membrane). After excitation, the formulated P680* (P680 excitation by the absorption of light to form P680*) donates the electrons through the electron transport chains for hydrogen concentration gradient construction and subsequently for adenosine triphosphate (ATP) biosynthesis, resulting in the simultaneous transformation of P680* to highly oxidative species (P680+). P680+ is extremely electronegative, capable of taking electrons from water molecules (water oxidation) and releasing oxygen molecules to restore the ground state P680 38. The O_2_ molecules are transferred through active diffusion to achieve oxidation of the microenvironment. At the same time, under the same laser irradiation, the photosensitizer (Ce6) was immediately activated to the singlet state (^1^Ce6) and then converted to the triplet state (^3^Ce6) 39. The triplet oxygen molecules (^3^O_2_) and (^3^Ce6) were produced by the TTA process to generate the ground state Ce6 Photosensitizer and strong oxidizing singlet oxygen (^1^O_2_) (Figure 2A).

*Syne* does not grow in the dark, but grows rapidly in the light (Figure S5), happily, Bac@Au-Ce6 also showed the same growth trend, (Figure 2B) which is important for the controlled growth of the engineered bacteria and the extensive application of follow-up. We next investigated the oxygen production of Bac@Au-Ce6 under different light conditions. Interestingly, Bac@Au-Ce6 produced the highest amount of oxygen under 660 nm laser irradiation, while the oxygen yield under LED irradiation was higher than that under 808 nm laser irradiation (Figure 2C). Compared with *Syne*, it was found that the load of Au-Ce6 did not affect the oxygen production capacity of *Syne* (Figure S6). Meanwhile, the results also show that the amount of oxygen produced is positively correlated with the amount of *Syne* in Bac@Au-Ce6 (Figure 2D). It is worth noting that it does not affect the production of O_2_ in a hypoxia environment, which provides favorable conditions for utilization in hypoxic TME Figure S7). These results indicate that the active Bac@Au-Ce6 engineered bacteria possess both photoautotrophic and photosynthetic oxygen production capabilities, effectively ensuring their biosafety as a novel oxygen production system. ROS production is crucial for realizing PDT efficacy. As shown in Figure 2E, 1,3-Diphenylisobenzofuran (DPBF) was used as a specific sensing probe for the detection of ^1^O_2_ in ROS. Compared to the control groups (DPBF+660/808 nm laser), the characteristic peak (λ = 417 nm) of DPBF declined progressively with the prolonged laser irradiation time subject to the treatment of “*Syne* (660 nm)” and “Au-Ce6 (660 nm)”, implying a sustained generation of ^1^O_2_. Furthermore, the group of “Bac@Au-Ce6 (660 nm)” after laser irradiation for 10 min, manifested the highest production of ^1^O_2_.

In the *Syne*, extremely weak fluorescence enhancement of SOSG can be observed in 15 min of laser irradiation (660 nm, 20 mW/cm^2^) (Figure 2F). In contrast, the additions of Au-Ce6 at concentrations of 3 µg/mL lead to a weak fluorescence enhancement (Figure 2G). In comparison, the SOSG fluorescence from Bac@Au-Ce6 intensities is correspondingly much higher than that of *Syne* and Au-Ce6 (Figure 2H). In 15 min of laser irradiation, Bac@Au-Ce6 eventually results in a relative fluorescence enhancement. These properties validate the robust ^1^O_2_ production performance of Bac@Au-Ce6. The promoted ^1^O_2_ generation is specifically attributed to the light-enabled photosynthetic oxygenation by the bacterial cells and the immediate oxygen activation by the photosensitizer. These features are believed to benefit the cellular and animal PDT process both *in vitro* and *in vivo*.

**Figure 3:**
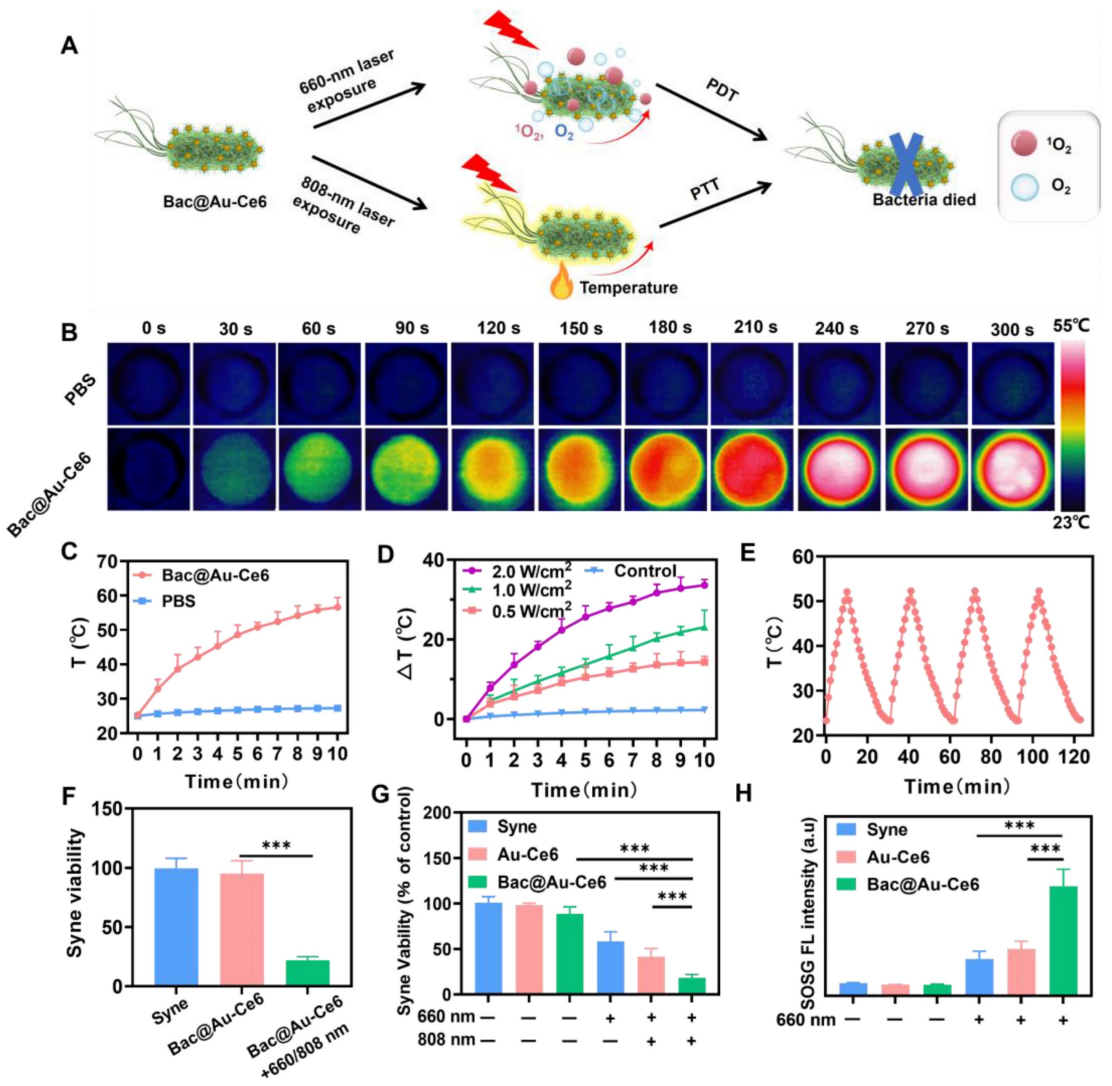
***in vitro* biological properties of Bac@Au-Ce6. (A)** Schematic diagram of the PTT and PDT effects of Bac@Au-Ce6. **(B)** Infrared thermal images of PBS and Bac@Au-Ce6 after irradiation. **(C)** Temperature profiles of Bac@Au-Ce6 and PBS under 808 nm laser irradiation (2 W cm^-2^, 10 min) (Mean ± SD, n = 3). **(D)** Temperature changes of Bac@Au-Ce6 under 808 nm laser irradiation with different power densities (0.5, 1, and 2 W cm^-2^, 10 min) (Mean ± SD, n = 3). **(E)** Temperature variation curves of Bac@Au-Ce6 followed by four cycles of irradiation. (F, G) The viability of *Syne* after different treatments (Mean ± SD, n = 3). **(H)** The ^1^O_2_ generation by *Syne*, Au-Ce6, or Bac@Au-Ce6 in a hypoxic condition with 660 laser irradiations (20 mW/cm^2^, 10 min), as determined by SOSG (ex/em: 488/525 nm) (Mean ± SD, n = 3).

Bac@Au-Ce6 possesses a PTT effect and enhanced PDT effect. The high temperature generated by the PTT effect and a large amount of ROS generated by the PDT effect Synergistically inhibited the viability of *Syne* bacteria. Provides controlled biosecurity for engineered bacteria (Figure 3A). To evaluate the PTT performance of Bac@Au-Ce6, Bac@Au-Ce6 was irradiated under 808 nm laser (2 W/cm^2^) for different times and thermal imaging images at specific time points were recorded with an infrared camera. According to Figure 3B and 3C, the temperature of Bac@Au-Ce6 increased rapidly with time after laser irradiation. In addition, the temperature increase is related to the laser irradiation power. (Figure 3D). No significant change in the PTT performance of Bac@Au-Ce6 was observed after four “heating/cooling” irradiation cycles (Figure 3E), demonstrating the desirable PTT stability of Bac@Au-Ce6.

To determine whether the presence of Bac@Au-Ce6 would affect bacterial viability, As shown in Figure 3F and 3G, the survival of *Syne* and Bac@Au-Ce6 were no difference, suggesting that Au-Ce6 did not affect bacterial growth. However, the number of bacteria was significantly reduced after laser irradiation (808 nm, 2 W/cm^2^, 10 min; 660 nm, 20 mW/cm^2^, 10 min), which may be the effect of ^1^O_2_ and the heat generated by Au NPs that exerted the antibacterial effect. Interestingly, Bac@Au-Ce6(+) had a more pronounced inhibitory effect on *Syne* than the addition of free Au-Ce6, providing a basis for better removal of bacterial residues *in vivo* after treatment and increased biosafety (Figure 3G). ^1^O_2_ generation after different treatments in an anoxic environment was measured with Singlet Oxygen Sensor Green (SOSG). As expected, laser irradiation (660 nm, 10 min) induced a small amount of ^1^O_2_ from Au-Ce6 under anoxic conditions, indicating that the PDT effect of Au-Ce6 was suppressed under anoxic conditions. Interestingly, exposure of *Syne* to laser irradiation (660 nm, 10 min) also produced a small amount of ^1^O_2_, which may have been converted from the oxygen produced by photosynthesis. Furthermore, compared to *Syne* and Au-Ce6, Bac@Au-Ce6 had significantly enhanced ^1^O_2_ generation under laser irradiation (660 nm, 10 min), indicating that photosynthesis improved the PDT effect of Bac@Au-Ce6 through *Syne* (Fig. 3H). The above results indicate that Bac@Au-Ce6 has PDT and PTT effects.

### Cellular Oxygenation and PTT-PDT of Bac@Au-Ce6

**Figure 4:**
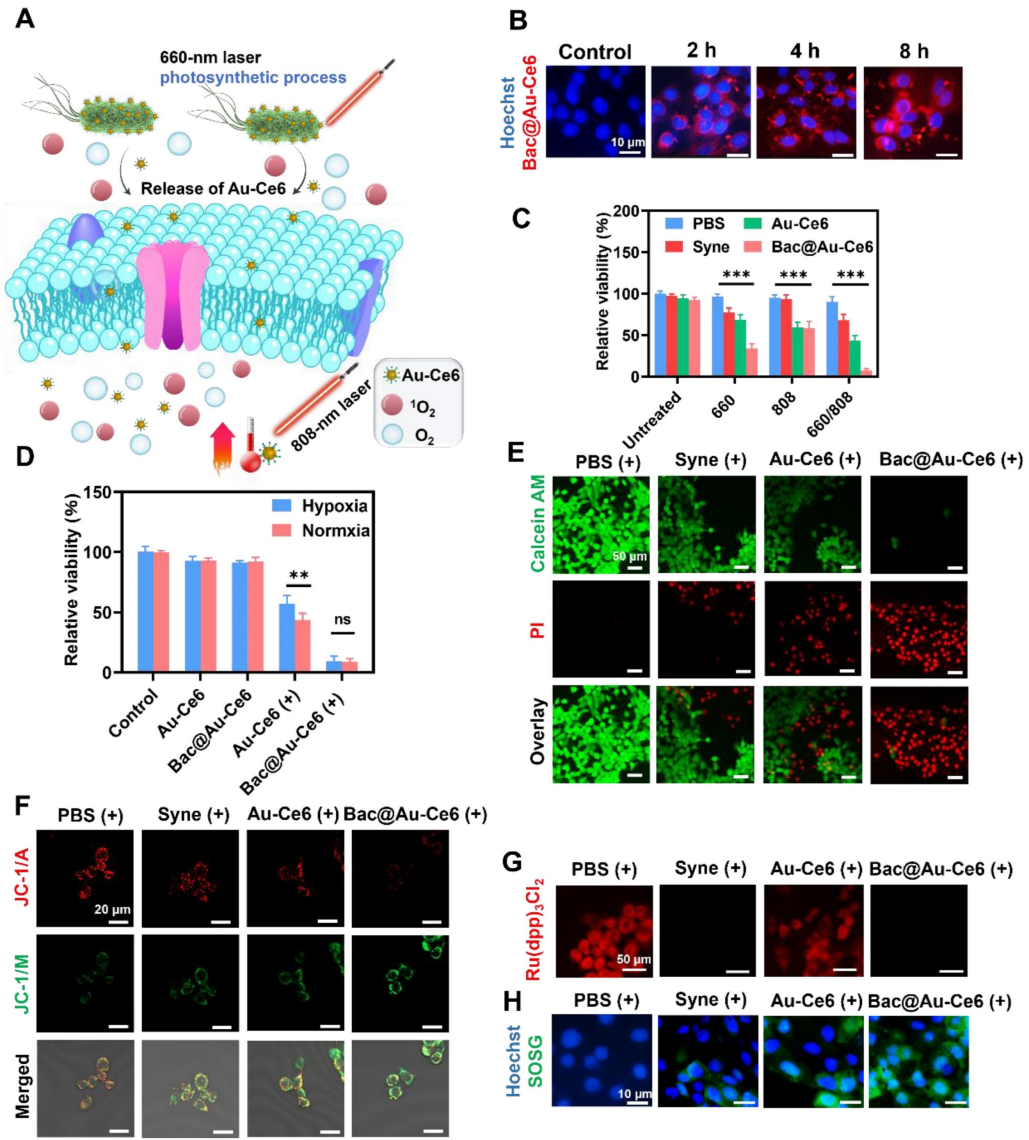
***In vitro* PDT and PDT performance. (A)** Schematic illustration of the PDT and PTT process using Bac@Au-Ce6 under laser irradiation (660 nm laser, 20 mW/cm^2^, 10 min; 808 nm laser, 2 W/cm^2^, 10 min). **(B)** *In vitro* cellular uptake of RhB-conjugated Bac@Au-Ce6, Scale bar = 10 μm. **(C)** Viability of 4T1 cells after various treatments (Mean ± SD, n = 3, ***P < 0.001). **(D)** Viability of 4T1 cells after treated with Au-Ce6, Bac@Au-Ce6, Au-Ce6 (+) and Bac@Au-Ce6 (+) (+:660 nm laser, 20 mW/cm^2^, 10 min; 808 nm laser, 2 W/cm^2^, 10 min) under normoxic or hypoxic conditions (Mean ± SD, n = 3). **(E)** Calcein AM/PI co-staining 4T1 cells in different treatments. Scale bars = 50 µm. **(F)** Confocal microscopic images of 4T1 cells stained with JC-1 dye after different treatments. scale bar = 20 μm. (+: 660 nm laser, 20 mW/cm^2^, 10 min, 808 nm laser, 2 W/cm^2^, 10 min). **(G)** Confocal microscopic images of 4T1 tumor cells stained by O_2_ indicator Ru(dpp)_3_Cl_2_ probe when treated with PBS (+), *Syne* (+), Au-Ce6 (+), Bac@Au-Ce6 (+) under a hypoxic conditions. (+: 660 nm laser, 20 mW/cm^2^, 10 min). **(H)** Confocal microscopic images of 4T1 tumor cells stained by SOSG probe when treated with PBS (+), *Syne* (+), Au-Ce6 (+), Bac@Au-Ce6 (+) (+: 660 nm laser, 20 mW/cm^2^, 10 min; 808 nm laser irradiation, 2 W cm^-2^, 10 min).

As the photosensitizers were adsorbed on the Bac@Au-Ce6 engineered bacteria, the Au-Ce6 release was investigated. It was determined that Au-Ce6 was released rapidly after laser irradiation, while it was released slowly without laser irradiation (Figure S8), which may be because the laser irradiation caused the death of the bacteria and promoted the shedding of Au-Ce6 from the surface of the bacteria. When Bac@Au-Ce6 engineered bacteria were delivered into cultures of murine mammary 4T1 tumor cells, Bac@Au-Ce6 distribution into tumor cells was observed. At 2 h of co-incubation, slight red fluorescence could be observed within the tumor cells, while the fluorescence became apparent within 4 h and increased after 8 h of co-incubation (Figure 4B). Specifically, Bac@Au-Ce6 modified with Rhb-exhibited red fluorescence. Distribution of Au-Ce6 into cells can be determined when tumor cell nuclei are stained with DAPI dye. These photosensitive Bac@Au-Ce6 engineered bacteria act as Au-Ce6 carriers to deliver photosensitizers and photothermal agents to tumor cells for potential photosynthetic oxygenation-enhancing cellular PDT and PTT under laser irradiation (Figure 4A).

Subsequently, the cytotoxicity of Bac@Au-Ce6 was investigated using 4T1 and L929 cells by the standard methyl thiazolyl tetrazolium (MTT) assay (Figure S9). The treated 4T1 and L929 cells maintained high viability even at feed ratios as high as 8 μg of Ce6 concentration/10^8^ *Syne* cells, validating the extraordinary biocompatibility. While under laser irradiation (20 mW/cm^2^, 10 min) to induce cellular oxygenation and PDT, the group of the *Syne*, Au-Ce6, and Bac@Au-Ce6, 4T1 tumor cells were killed (22.6%), (31.3%), (65.5%), respectively. In addition, the PTT process of the Au-Ce6 and Bac@Au-Ce6 treatment groups also showed a certain anti-4T1 tumor cell effect under laser irradiation (808 nm, 2 W/cm^2^, 10 min) (40.2%), (41.6%). When the Bac@Au-Ce6 group was irradiated with laser light (660 nm, 20 mW/cm^2^, 10 min; 808 nm, 2 W/cm^2^, 10 min), the induced PDT and PTT together produced massive 4T1 cell death (Figure 4C). Besides, the Bac@Au-Ce6 was treated by laser irradiation under normoxic and hypoxic conditions and the PDT effect in the two environments was compared by MTT method (Figure 4D). The results showed that the PDT effect of Au-Ce6(+) is limited in hypoxia conditions. However, there was no significant difference in the viability of 4T1 cells treated with Bac@Au-Ce6 (+) (660 nm, 20 mW/cm^2^, 10 min; 808 nm, 2 W/cm^2^, 10 min) in hypoxic or normoxic environments, indicating that Bac@Au-Ce6 could provide sufficient O_2_ for PDT-triggered ^1^O_2_ production. From the confocal images of 4T1 tumor cells stained with live/dead fluorescence kits after treatment respectively, it can be further revealed that after laser irradiation (660 nm laser, 20 mW/cm^2^, 10 min; 808 nm laser, 2 W/cm^2^, 10 min) antitumor activity of *Syne*, Au-Ce6, and Bac@Au-Ce6 (Figure 4E). Cells treated with PBS and PBS (+) both showed strong green fluorescence, combined with previous MTT results, indicating that only laser irradiation did not affect the cells. In addition, cells treated with *Syne*, Au-Ce6, and Bac@Au-Ce6 respectively, showed strong green staining, indicating a negligible effect on cells (Figure S10A). After irradiation, the Bac@Au-Ce6 group showed most of the red fluorescent cells, indicating that most of the tumor cells had died. However, insufficient cellular PDT effect was observed in the Au-Ce6-treated group after the same laser irradiation, indicating that *Syne*'s oxygenation effectively promoted the production of singlet oxygen, resulting in more significant cell death. The green fluorescence of JC-1 represents mitochondrial damage, as shown in Figure 4F, Bac@Au-Ce6 after laser irradiation showed the strongest green fluorescence. The obtained results were consistent with the Live/dead staining results.

The engineered bacteria can lead to increased cellular oxygen levels. To verify this, the intracellular hypoxia indicator [Ru(dpp)_3_]Cl_2_ was used to visualize the O_2_ production process 40. 4T1 tumor cells were initially subjected to 12 h of hypoxia-sealed conditions, followed by co-incubation and treatment with Au-Ce6 and Bac@Au-Ce6. It was observed from confocal microscopy images that both untreated and Au-Ce6 (660 nm, 10 min, 20 mW/cm^2^) treated tumor cells displayed strong intracellular red fluorescence, indicating that 4T1 tumor cells had relatively low pO_2_ values after O_2_ deprivation preincubation (Figure 4G). Au-Ce6 (660 nm, 10 min, 20 mW/cm^2^), PBS, *Syne*, Au-Ce6, and Bac@Au-Ce6 treatment did not alter the hypoxic state of tumor cells (Figure S10B). However, the intracellular fluorescence of [Ru(dpp)_3_]Cl_2_ was significantly attenuated when treated with *Syne* and Bac@Au-Ce6 engineered bacteria at 660 nm laser (20 mW/cm^2^) for 10 min, indicating that by co-incubating with *Syne* cells effective *in vitro* oxygenation is achieved. Under sufficient O_2_, the PDT effect of photosensitizers can be effectively enhanced.

Next, the intracellular ^1^O_2_ species generation was visualized by the SOSG probe. After co-incubating with *Syne*, Au-Ce6, and Bac@Au-Ce6, respectively, the above groups were subjected to laser irradiation (660 nm, 20 mW/cm^2^, 10 min), and then the fluorescence of treated 4T1 tumor cells was observed by confocal microscope. In 4T1 tumor cells, with *Syne* (660 nm, 10 min, 20 mW/cm^2^) or Au-Ce6 (660 nm, 10 min, 20 mW/cm^2^), very weak SOSG fluorescence can be observed, indicating a small amount of ^1^O_2_ produced in the absence or low dose of Au-Ce6 (Figure 4H). While in Bac@Au-Ce6 (660 nm, 10 min, 20 mW/cm^2^) treated cells, bright intracellular SOSG fluorescence could be observed, indicating that significant ^1^O_2_ was generated through cascaded photosynthesis and photodynamics. It is worth mentioning that no green fluorescence was observed under PBS, Syne, Au-Ce6, and Bac@Au-Ce6 treatments indicating no singlet oxygen generation (Figure S10C). In summary, Bac@Au-Ce6 engineered bacteria enhance PDT production of ^1^O_2_ through sustained photosynthetic oxygen release, which can offer promising potential for tumor PDT at relatively low laser power density (20 mW/cm^2^).

### Bac@Au-Ce6 targeting to improve tumor hypoxia

As solid tumors are typically negatively charged 41, the resulting positive charge on Bac@Au-Ce6 meant they were electrostatically attracted to the tumor sites. In addition, due to its micron size, *Syne* is theoretically easy to be captured by tumor capillaries, which is expected to achieve targeting of tumors 42, 43.

**Figure 5:**
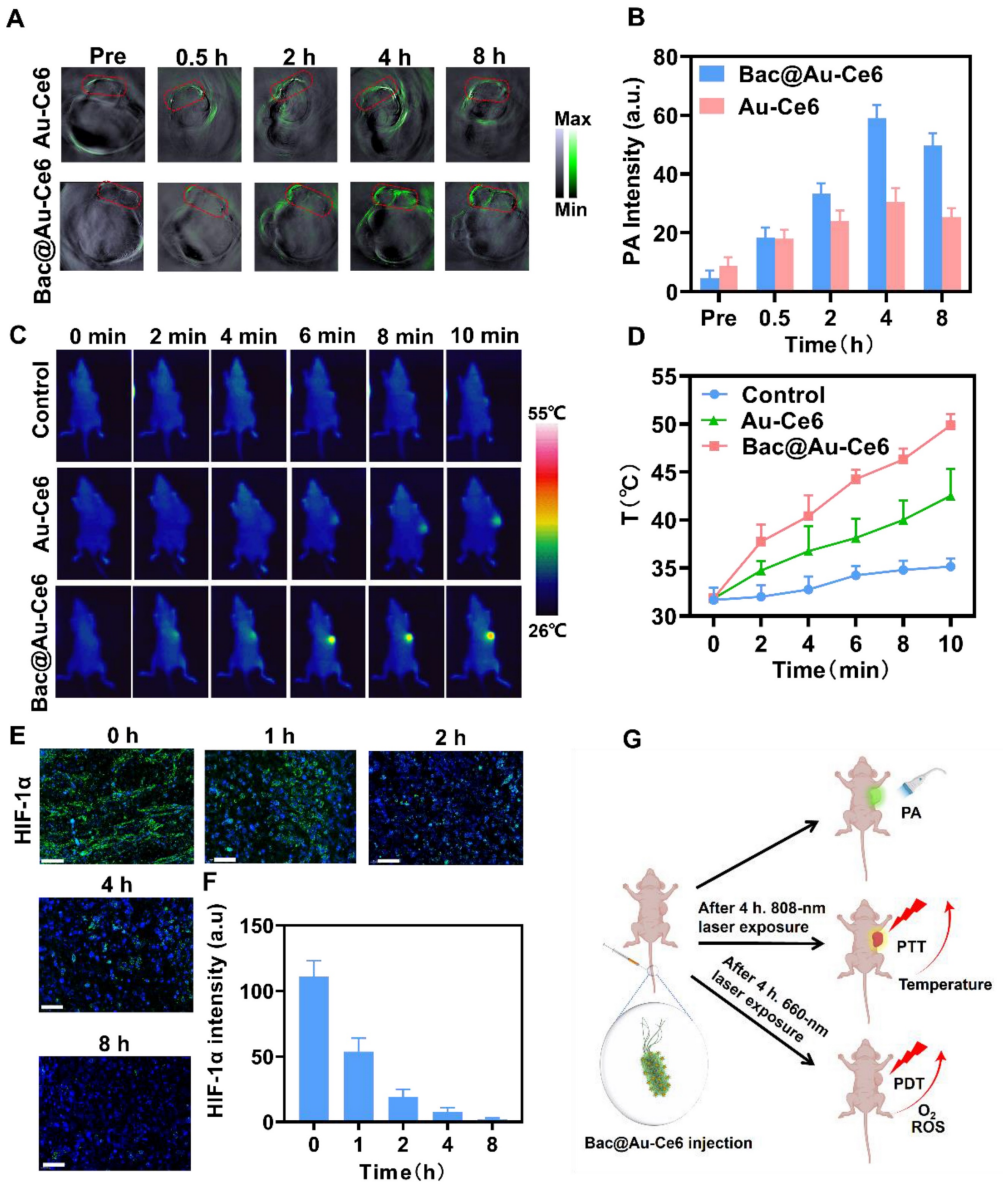
***In vivo* biological properties of Bac@Au-Ce6. (A)** *In vivo* PA images of tumorous tissue at different time points after i.v. injection of Bac@Au-Ce6. **(B)** Average PA intensity of tumor site corresponding to **(A)** (Mean ± SD, n = 5). **(C)** Thermographic mapping of BALB/c mice bearing 4T1 tumors after i.v. injection of Bac@Au-Ce6 for 4 h and subject to 808 nm laser irradiation for up to 10 min. **(D)** Temperature elevation of tumor region corresponding to **(C)** (Mean ± SD, n = 5). **(E)** Representative HIF-1α staining of tumor sections at different times. **(F)** The corresponding immunohistochemical index quantitative analysis in **(E)** (Mean ± SD, n = 5). **(G)** Schematic diagram of *in vivo* PA imaging, PDT, and PTT effect of Bac@Au-Ce6.

To explore the tumor-targeted delivery efficacy of Bac@Au-Ce6, tumor-bearing mice were i.v. injected with Bac@Au-Ce6 or Au-Ce6. PA images were taken at 0, 0.5, 2, 4, and 8 h after injection (Figure 5A). The results showed that the high-contrast PA signal detected at the tumor site gradually increased with time (Figure 5B). The PA signal of Bac@Au-Ce6 was more concentrated at the tumor site than that of Au-Ce6, and reached a peak at 4 h after administration, indicating the highest concentration of Bac@Au-Ce6 at the tumor site. In addition, *in vivo* fluorescence imaging was also used to detect the targeting of Bac@Au-Ce6. The results showed that Au-Ce6 fluorescence could effectively accumulate in the tumor site within 4 h, which may be depended on the EPR effect. As compared with Au-Ce6 without modifications, Bac@Au-Ce6 showed much higher accumulation in tumors. It is known that fluorescence imaging penetrates tissue less than 1 cm and has low resolution 44. We collected tissues and performed *in vitro* fluorescence imaging to further verify this. The fluorescence intensity of Bac@Au-Ce6 and Au-Ce6 tumor tissues is higher than that of other organs, and Bac@Au-Ce6 has a stronger fluorescence signal at the tumor site than Au-Ce6 (Figure S11). Time-dependent biological distributions of Au-Ce6 and Bac@Au-Ce6 were analyzed by ICP-MS (Figure S12). It can be seen that Bac@Au-Ce6 is more abundant in the tumor site than Au-Ce6 and less abundant in other organs in the body. All the above results prove that Bac@Au-Ce6 has good targeting properties. The temperature increase in the tumor region triggered by 808 nm laser irradiation for 10 min was monitored 4 h after i.v. injection of Bac@Au-Ce6. A thermal imager was used to dynamically record thermal images of the mouse's body at specific time points during laser irradiation. Compared with mice injected with PBS and Bac@Au-Ce6, the local temperature of the tumor site in the Bac@Au-Ce6 group increased rapidly with the laser irradiation time, and the final average temperature reached 49.8 °C (Figure 5C and 5D).

Mice treated with laser irradiation (660 nm laser, 20 mW/cm^2^, 10 min; 808 nm laser, 2 W/cm^2^, 10 min) dissected tumors and found that HIF-1α levels were significantly reduced (Figure 5E and 5F), indicating that tumor hypoxia was relieved after treatment. Overall, Bac@Au-Ce6 can reach the tumor site by i.v. injection, and can exert precise therapeutic effects by PA imaging. PTT and enhanced PDT can be induced at tumor sites after different laser irradiation (Figure 5G).

### *In vivo* antitumor effects of Bac@Au-Ce6

**Figure 6:**
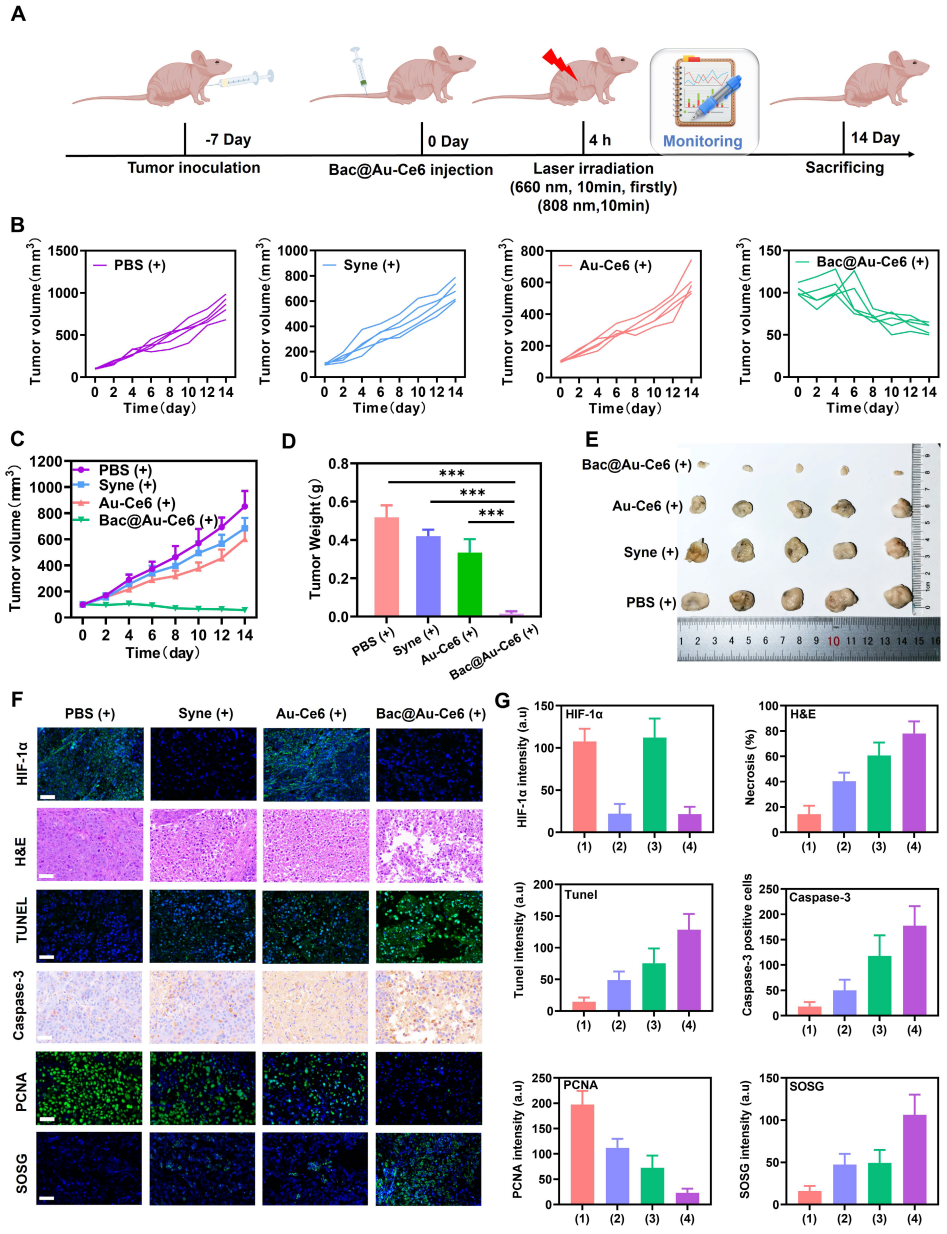
***In vivo* antitumor activity of Bac@Au-Ce6. (A)** Schematic diagram of treatment of 4T1 tumor-bearing mice. **(B)** Tumor growth curves of individual mice after different treatments. **(C)** Average tumor growth curves of all the groups (Mean ± SD, n = 5). **(D)** The tumor weights at 14 d in different groups (Mean ± SD, n = 5). **(E)** Representative photographs of tumors from different groups after 14-day therapy. **(F)** Histological analysis of tumor tissues, stained with HIF-1α for hypoxia, PCNA for cell proliferation, SOSG for ^1^O_2_, H&E for inflammation, TUNEL and Caspase-3 for apoptosis and **(G)** corresponding index quantitative analysis after 14-day therapy. Scale bar: 100 µm. (1):PBS(+), (2):*Syne* (+), (3):Au-Ce6(+), (4):Bac@Au-Ce6(+) (+: 660 nm laser, 20 mW/cm^2^, 10 min, firstly; 808 nm laser irradiation, 2 W cm^-2^, 10 min).

We next assessed the efficacy of Bac@Au-Ce6 engineered bacteria in enhancing PDT in mice inoculated with 4T1 breast cancer cells (detailed experimental designs are shown in Figure 6A). When the size of tumors reached about 100 mm^3^ in volume (about 7 days), Firstly, the effects of PBS, *Syne*, Au-Ce6 and Bac@Au-Ce6 on tumor sites were evaluated. After intravenous injection of *Syne*, Au-Ce6, and Bac@Au-Ce6, there was no significant inhibitory effect on tumor growth compared with the PBS group. In addition, there was no significant difference in tumor size between PBS(+) group and the PBS group, indicating that laser irradiation alone had no significant effect on tumor growth (Figure S13A-S13C). The body weight of mice was in the normal range and there was no significant difference in different histological analyses (Figure S13D-S13F). After i.v. injection of Bac@Au-Ce6, the tumors were sequentially irradiated by 660-nm laser (20 m W/cm^2^, 10 min, firstly) and 808-nm laser (2 W/cm^2^, 10 min). Bac@Au-Ce6-directed PTT-PDT combination therapy strongly inhibited tumor growth, whereas Au-Ce6-directed PTT-PDT therapy resulted in approximately 48% tumor inhibition (Figure 6B-6E). This suggests that the *Syne* enhances PDT production of ^1^O_2_ through sustained photosynthetic oxygen release. Immunohistochemical staining was performed on tumor tissues after different treatments further to evaluate the antitumor effect of Bac@Au-Ce6 *in vivo*. The staining results showed that HIF-1α expression was significantly decreased after Bac@Au-Ce6 with laser therapy, indicating that the oxygen content at the tumor site was increased, thereby significantly improving the treatment efficiency of PDT. PCNA staining showed that compared with other groups, Bac@Au-Ce6 with laser significantly reduced PCNA-positive cells in tumor tissue, indicating that it could significantly inhibit tumor proliferation. In addition, SOSG, H&E, caspase-3, and TUNEL staining confirmed that the Bac@Au-Ce6 after laser irradiation could significantly increase the ^1^O_2_ level in tumor tissue, thereby inducing tumor tissue necrosis and apoptosis, and exerting the Strong anti-tumor effect. (Figure 6F and 6G).

### Biosafety Evaluation of Bac@Au-Ce6

**Figure 7:**
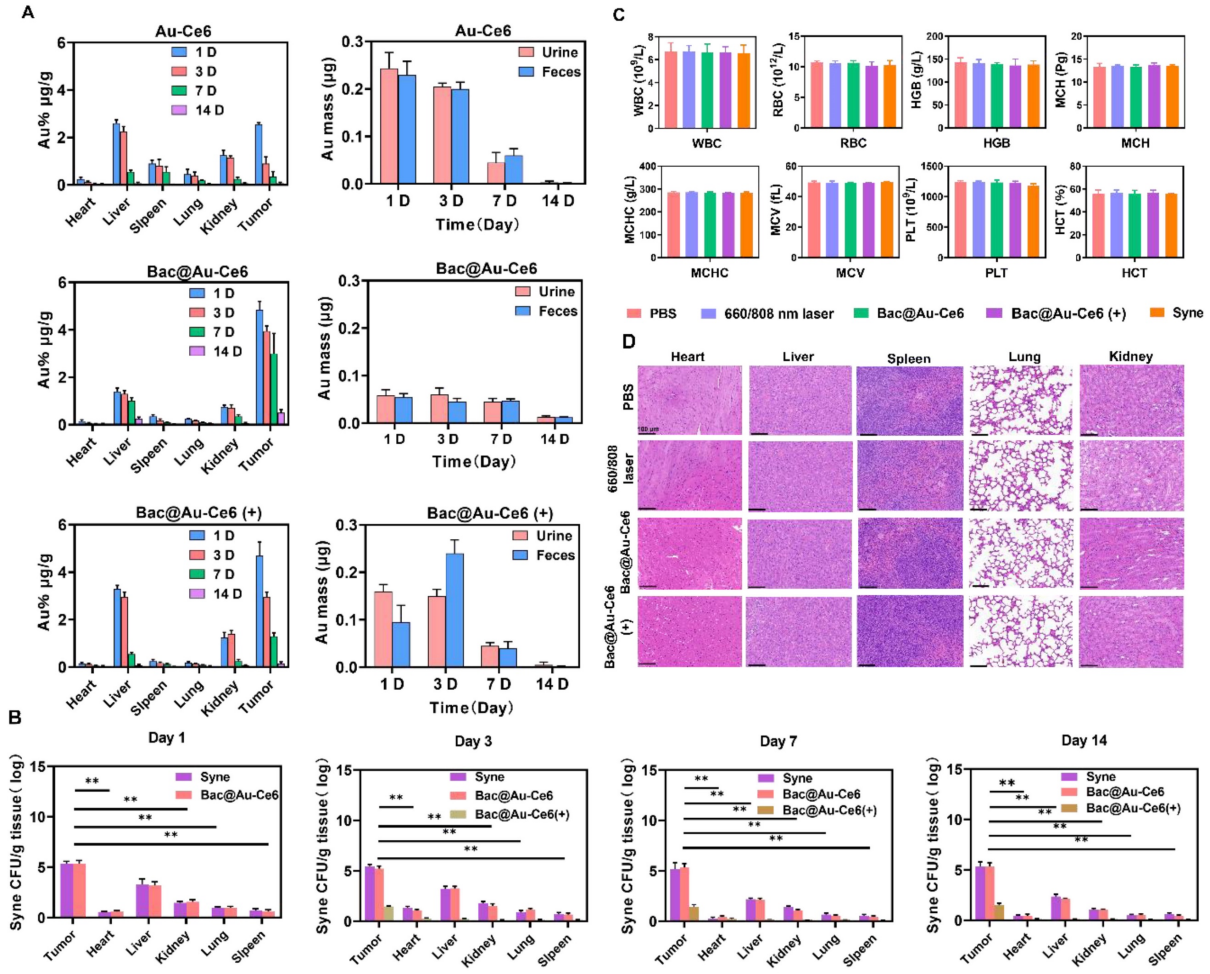
***In vivo* Safety evaluation of Bac@Au-Ce6**. **(A)** Bio-distribution of Au-Ce6, Bac@Au-Ce6, and Bac@Au-Ce6 (+) in major organs and tumor and Au contents in urine and feces of mice within 14 d (Mean ± SD, n = 5). **(B)** Statistical analysis of Syne, Bac@Au-Ce6, and Bac@Au-Ce6 (+) in organs, over time (Mean ± SD, n = 5, **P < 0.01). **(C)** Blood routine and blood biochemistry tests of the mice 60 days after i.v. injection of various treatments (Mean ± SD, n = 5). WBC, white blood cells; RBC, red blood cells; HGB, hemoglobin; MCH, mean corpuscular hemoglobin; MCHC, mean corpuscular hemoglobin concentration; MCV, mean cell volume; PLT, blood platelet; HCT, hematocrit. **(D)** H&E staining images of the major organs (Heart, Liver, Spleen, Kidney, and Lung) of the mice at 14 days after different treatments (Mean ± SD, n = 5). Scale bar = 100 μm. (+: 660 nm laser, 20 mW/cm^2^, 10 min, firstly; 808 nm laser irradiation, 2 W cm^-2^, 10 min).

The *in vivo* clearance behaviors were studied. First, the time-dependent biological distribution of Au-Ce6, Bac@Au-Ce6, and Bac@Au-Ce6 (+) were analyzed by ICP-MS (Figure 7A). It can be seen that the content of Au in the body gradually decreased with the extension of time, and almost no accumulation was detected in the main organs 14 days after injection, suggesting that Au-Ce6, Bac@Au-Ce6, and Bac@Au-Ce6 (+) can be metabolized in the body (Figure 7A). In addition, we found that Au-Ce6 accumulated more at the tumor site through *Syne* delivery and was retained for a longer period. It is worth noting that Bac@Au-Ce6 after laser irradiation, the content of Au decreases rapidly, indicating that it can be metabolized from the body more quickly. It may be that the bacteria were affected by PTT and PDT, and their activity decreased or even died, leading to a faster release of Au-Ce6. Afterward, we explored the metabolic pathway of Au-Ce6, Bac@Au-Ce6, and Bac@Au-Ce6 (+) by carefully monitoring the changes of Au content in urine and feces of mice (Figure 7A). With the prolongation of the observation time, the total levels of Au detected in urine and feces gradually increased, strongly evidencing that Au-Ce6 could be excreted through the kidney and liver. Since bacteria release Au-Ce6, the *Syne* in these tissues was quantitated by counting bacterial colonies. The number of* Syne* colonies in tumors did not change significantly on the 14th day, but the concentration in all the major organs decreased, which showed that bacteria can be efficiently metabolized in major organs. It is worth noting that after laser irradiation treatment, the viability of *Syne* was greatly reduced in major organs and tumors (Figure 7B), which further enhances biosafety.

Importantly, the body weight of mice fluctuated within the normal range during the treatment process effects (Figure S14 and S15). In addition, various blood tests were within the normal range (Figure 7C), and H&E staining results showed that the heart, liver, spleen, lung, and kidney in each treatment group were indistinguishable from normal mice (Figure 7D), indicating the good biocompatibility of Bac@Au-Ce6. Besides, Survival analysis showed that 75% of mice receiving Bac@Au-Ce6 with laser therapy were still alive after 42 days of the experimental period, while all mice in the other experimental groups died (Figure S16), indicating that Bac@Au-Ce6 with laser therapy can significantly prolong the survival of 4T1 mice. Therefore, Bac@Au-Ce6 with 660 nm and 808 nm laser is an effective antitumor strategy, which has the advantages of good biocompatibility and enhanced antitumor efficacy.

## Conclusion

In conclusion, we developed a novel strategy to fabricate active photosynthetic bacteria/photosensitizer/photothermal agent hybrids for photosynthetic tumor oxygenation and PDT and PTT tumor therapy under different laser irradiation sources. Photosynthetic bacteria combined with Ce6 photosensitizer and Au NPs photothermal agent, the obtained Bac@Au-Ce6 effectively targets tumor tissues and further enhances the tumor accumulation of Au-Ce6. Meanwhile, Bac@Au-Ce6 continuously produced oxygen through photosynthesis under 660 nm laser irradiation, significantly improving the tumor hypoxia state and enhancing the PDT effect. In addition, the PTT effect activated by Bac@Au-Ce6 under 808 nm laser irradiation further enhanced the tumor lethality. The present work provides an efficient oxygenation strategy to enhance PDT. In addition, this strategy also provides an innovative idea for microbial-based engineered bacteria for multifunctional cooperative tumor therapy.

## Supplementary Material

Supplementary figures.
