# Supplementary material for: Light-triggered photosynthetic engineered bacteria for enhanced-photodynamic therapy by relieving tumor hypoxic microenvironment
Source: Theranostics. 2023 Mar 13;13(5):1632–48. doi: 10.7150/thno.81718 (PMC10086205; doi:10.7150/thno.81718)
Supplement: Supplementary file 1 — Supplementary figures. [file thnov13p1632s1.pdf]

Supplementary Information

For

Light-triggered photosynthetic engineered bacteria for enhanced-photodynamic therapy by relieving tumor hypoxic microenvironment

Chenyang Yin[1,#], ZeKun Wang[1,#], Chunxue Dai[1], Bangjia Yang[1], Weiyun Wang[1], Endong Yang[1], Feng Guo[1], Cundong Fan[2,*], Pu Zhang[3,*], Jikui Sun[4,*], Dongdong Sun[1,*]

[1] School of Life Sciences, Anhui Agricultural University, Hefei 230036, China

[2] Shandong Key Laboratory of TCM Multi-Target Intervention and Disease Control, the Second Affiliated Hospital of Shandong First Medical University, Taian, Shandong 271000, China

[3] Department of Cardiology, Affiliated Taian City Central Hospital of Qingdao University, Taian, Shandong, 271000, China

[4] Department of Neurosurgery, the First Affiliated Hospital of Shandong First Medical University & Shandong Provincial Qianfoshan Hospital, Jinan, China

[#] These authors contributed equally to this work.

***Corresponding authors:**

Dongdong Sun, Email: sunddwj@126.com; **Cundong Fan**, Email: tcdfan66@163.com; **Jikui Sun**, Email: jikuisun2015@163.com; **Pu Zhang**, Email: zp8198423@163.com

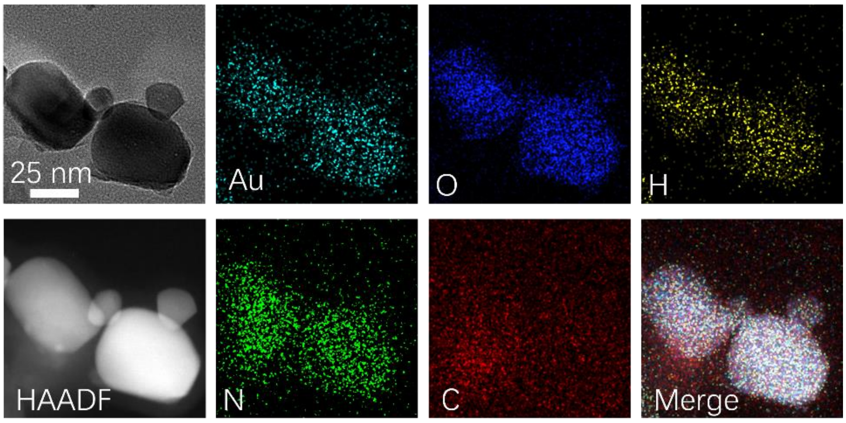

Figure S1. The TEM mapping of Au-Ce6. Scale bar: 25 nm.

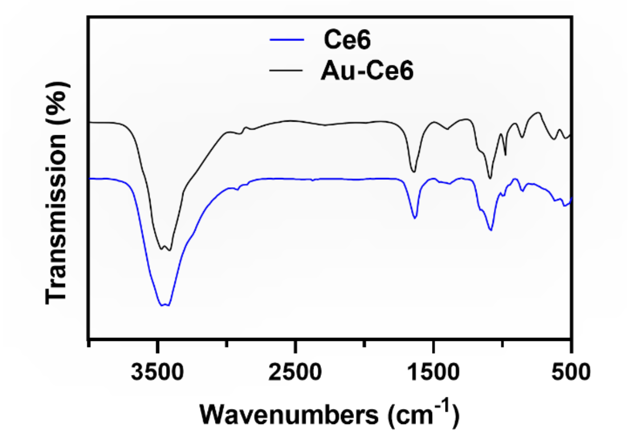

Figure S2. FTIR spectra of Ce6 and Au-Ce6.

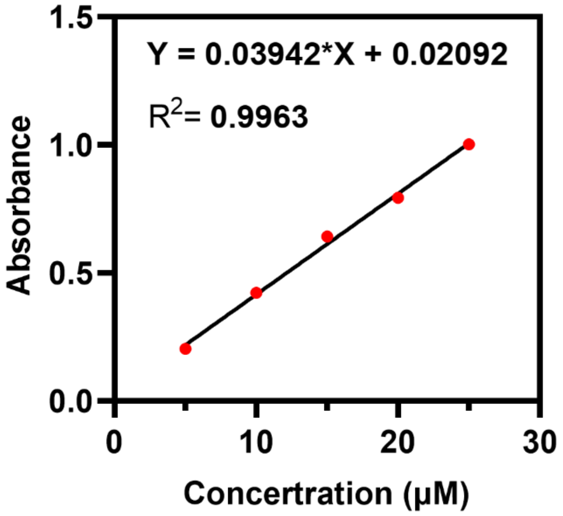

Figure S3. The standard curve for Ce6.

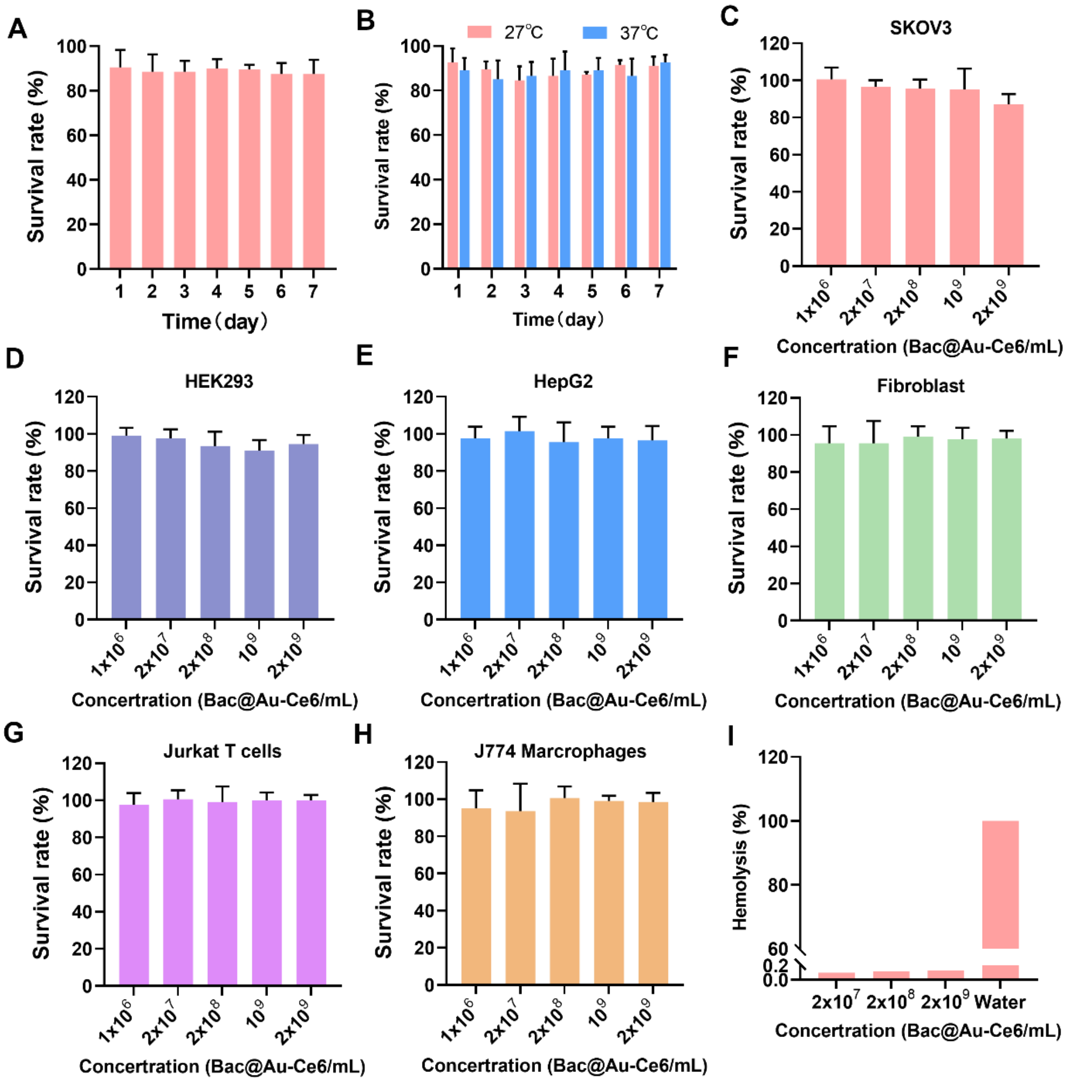

Figure S4. (A) Time-dependent survival of Bac@Au-Ce6 over 7 days. (B) Time-dependent survival of Bac@Au-Ce6 under growth conditions at 27°C and 37°C. (C-H) Cell viability of SKOV3, HEK293, HepG2, fibroblasts, Jurkat T, and J774 cells treated with Bac@Au-Ce6 (Syne = different concentrations, 3 μg ~ 30 μg of Ce6 concentration). (I) Quantification of percent hemolysis. Data are mean ± SD, n = 3.

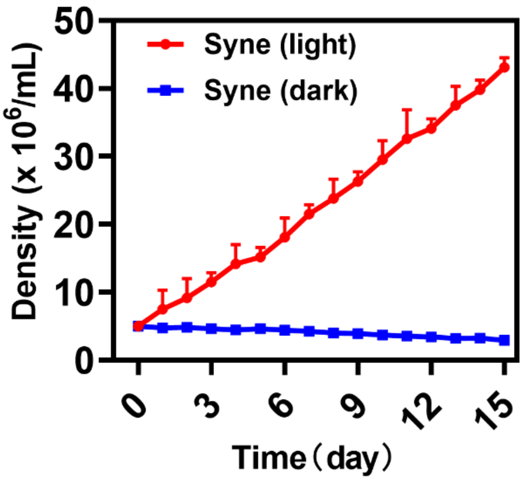

Figure S5. The growth curve of Syne in the light or dark (Mean ± SD, n = 3).

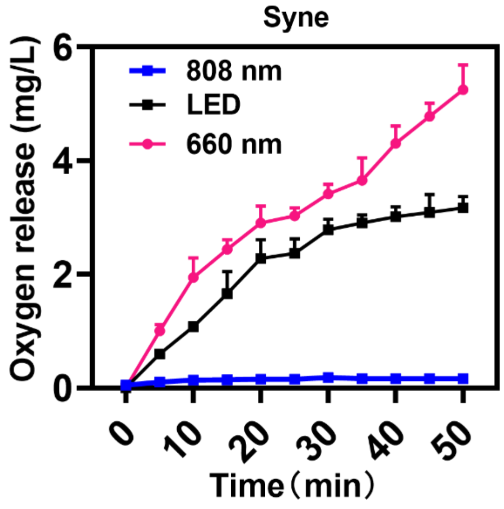

Figure S6. Oxygen production curves of Bac@Au-Ce6 under different conditions. LED irradiation with a wavelength range of 400–750 nm (Mean ± SD, n = 3).

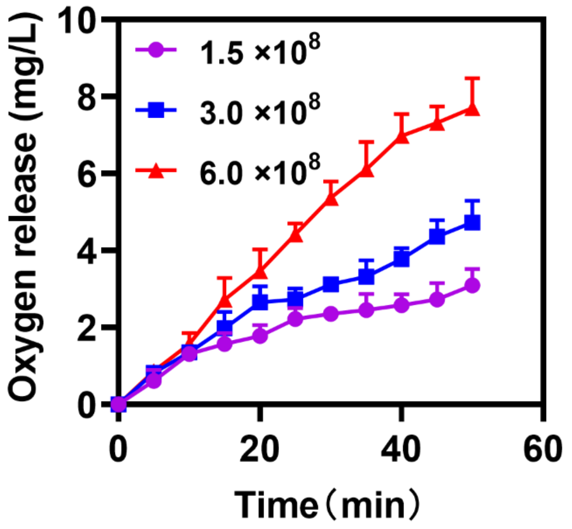

Figure S7. Oxygen production curves of various concentrations of Bac@Au-Ce6 treated under hypoxia with 660 nm laser irradiation (Mean ± SD, n = 3).

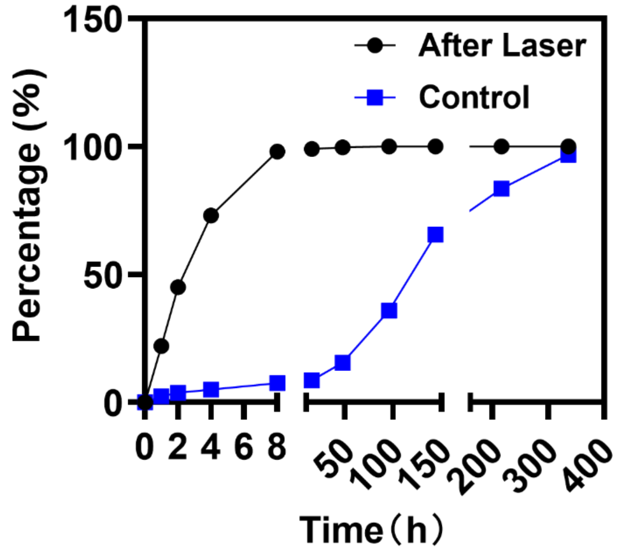

Figure S8. The release percentage of Ce6 versus time in Bac@Au-Ce6 (Laser: 660 nm, 20 mW/cm^2, 5 min, and 808 nm, 2 W/cm^2, 5 min).

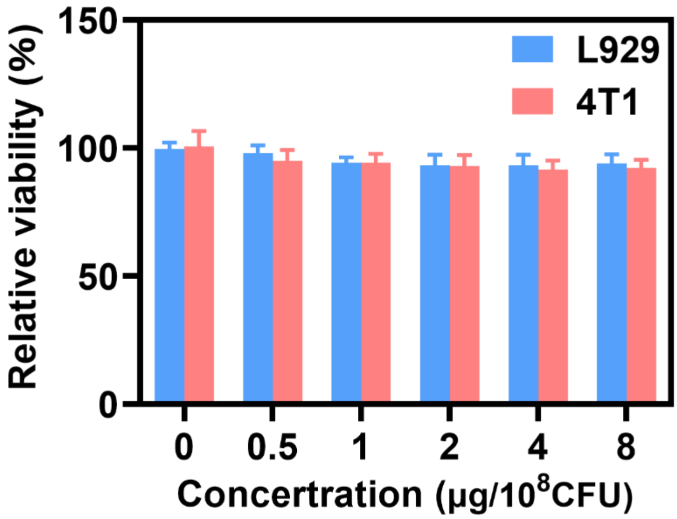

Figure S9. Cytotoxicity of Bac@Au-Ce6 on L929 and 4T1 cells (Mean ± SD, n = 3).

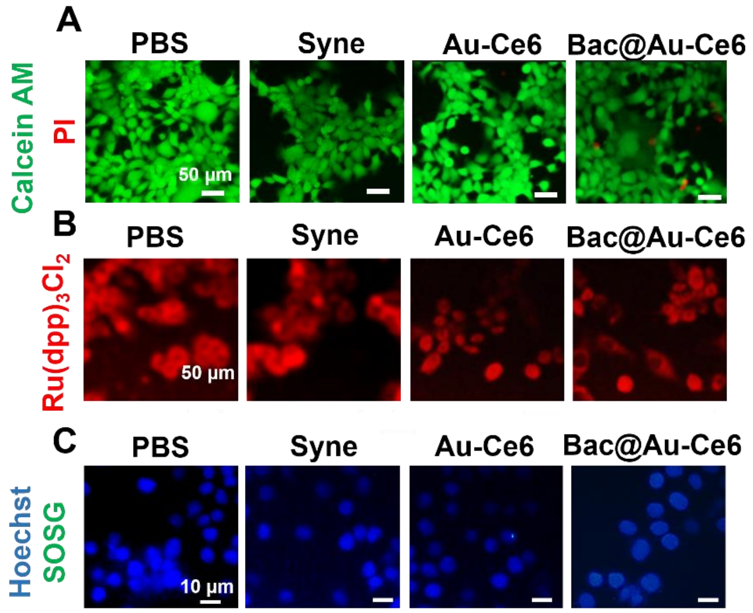

Figure S10. (A) Calcein AM/PI co-staining 4T1 cells in different treatments. Scale bars = 50 μm. (B) Confocal microscopic images of 4T1 tumor cells stained by O_2 indicator $Ru(dpp)_3Cl_2$ probe when treated with PBS, *Syne*, Au-Ce6, Bac@Au-Ce6. Scale bars = 50 μm. (C) Confocal microscopic images of 4T1 tumor cells stained by SOSG probe when treated with PBS, *Syne*, Au-Ce6, Bac@Au-Ce6. Scale bars = 10 μm.

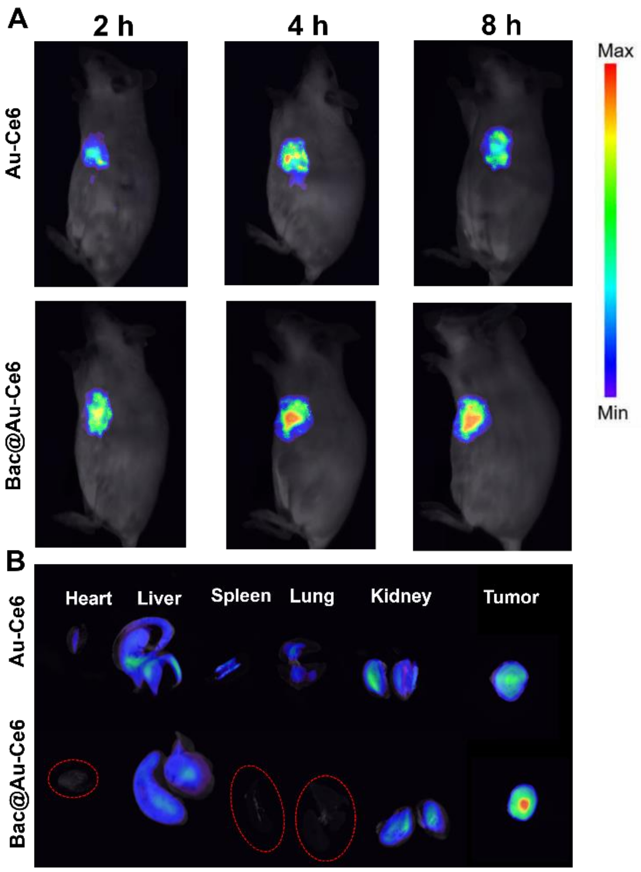

Figure S11. (A) In vivo fluorescence images of 4T1 tumor-bearing mice and (B) Fluorescence images of the major organs and tumors after the injection for 8 h.

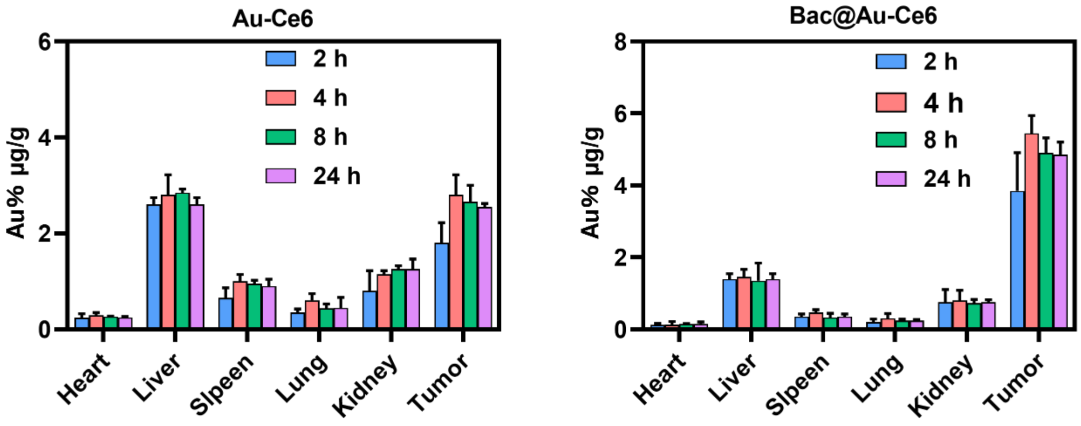

Figure S12. Bio-distribution of Au-Ce6, Bac@Au-Ce6, and Bac@Au-Ce6 in major organs and tumors within 24 h (Mean ± SD, n = 5).

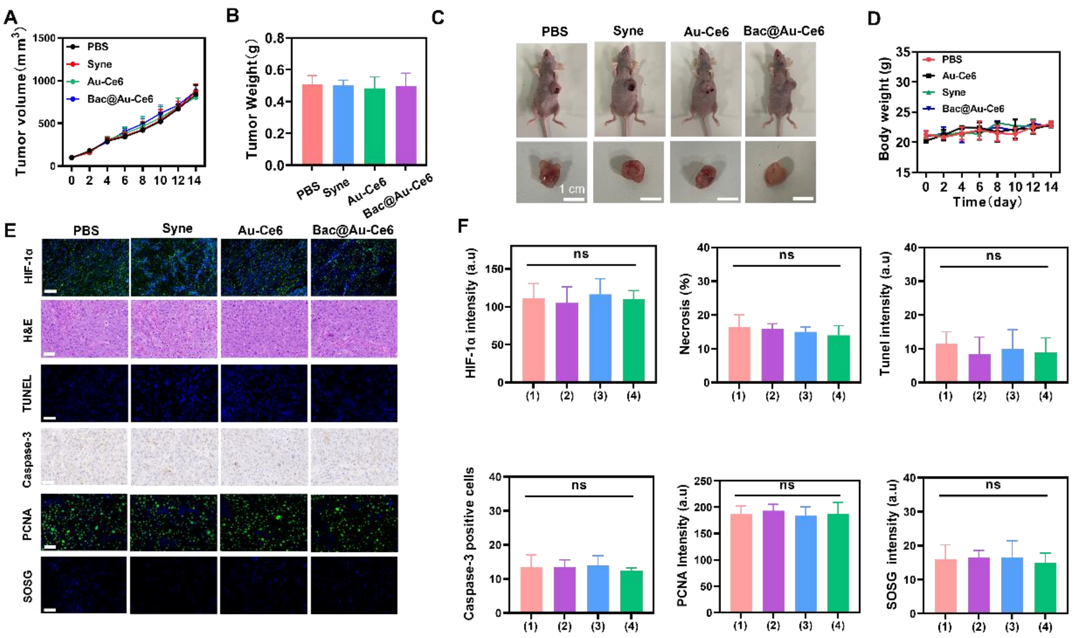

Figure S13. (A) Average tumor growth curves of all the groups (Mean ± SD, n = 5). (B) The tumor weights at 14 d in different groups (Mean ± SD, n = 5). (C) Representative photographs of mice and tumors from different groups after 14-day therapy. (D) The body weight of mice corresponds to different time points (Mean ± SD, n = 5). (E) Histological analysis of tumor tissues, stained with HIF-1α for hypoxia, PCNA for cell proliferation, SOSG for $^1O_2$, H&E for inflammation, TUNEL and Caspase-3 for apoptosis and (F) corresponding index quantitative analysis after 14-day therapy (Mean ± SD, n = 5). Scale bar: 100 μm. (1): PBS as control, (2): Syne, (3): Au-Ce6, (4): Bac@Au-Ce6.

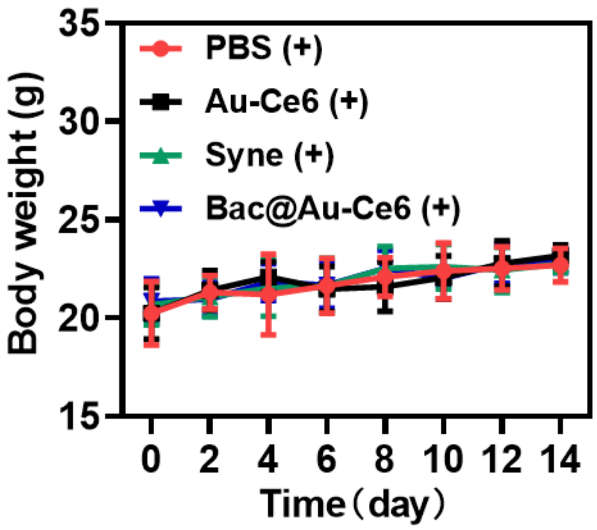

Figure S14. The body weight of mice corresponds to different time points (Mean ± SD, n = 5).

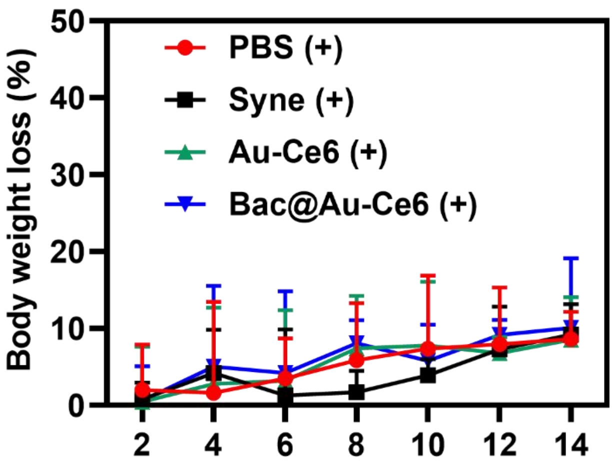

Figure S15. The percentage of “Body weight loss” [(starting weight - body weight at day 2, 4, 6, 8, 10, 12, 14) / starting weight] × 100% (Mean ± SD, n = 5).

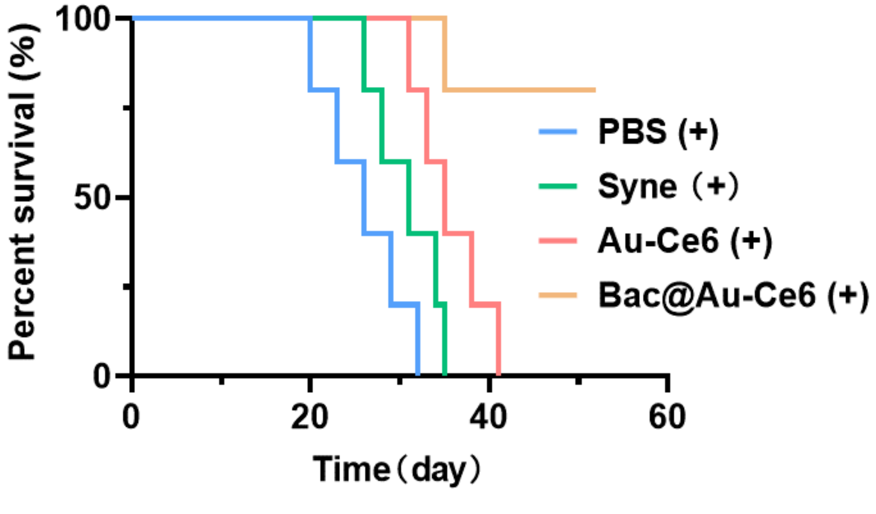

Figure S16. The survival rate of mice corresponds to different time points.
